# Constraints on upper crustal fluid circulation and seismogenesis from in-situ outcrop quantification of complex fault zone permeability

**DOI:** 10.1038/s41598-023-32749-4

**Published:** 2023-04-05

**Authors:** M. Curzi, F. Giuntoli, G. Vignaroli, G. Viola

**Affiliations:** Dipartimento di Scienze Biologiche, Geologiche ed Ambientali-BiGeA, Università degli studi di Bologna, Via Zamboni 67, 40126 Bologna, Italy

**Keywords:** Structural geology, Tectonics

## Abstract

The permeability of fault zones plays a significant role on the distribution of georesources and on seismogenesis in the brittle upper crust, where both natural and induced seismicity are often associated with fluid migration and overpressure. Detailed models of the permeability structure of fault zones are thus necessary to refine our understanding of natural fluid pathways and of the mechanisms leading to fluid compartmentalization and possible overpressure in the crust. Fault zones commonly contain complex internal architectures defined by the spatial juxtaposition of “brittle structural facies” (BSF), which progressively and continuously form and evolve during faulting and deformation. We present the first systematic in-situ outcrop permeability measurements from a range of BSFs from two architecturally complex fault zones in the Northern Apennines (Italy). A stark spatial heterogeneity of the present-day permeability (up to four orders of magnitude) even for tightly juxtaposed BSFs belonging to the same fault emerges as a key structural and hydraulic feature. Insights from this study allow us to better understand how complex fault architectures steer the 3D hydraulic structure of the brittle upper crust. Fault hydraulic properties, which may change through space but also in time during an orogenesis and/or individual seismic cycles, in turn steer the development of overpressured volumes, where fluid-induced seismogenesis may localize.

## Introduction

The internal architecture of fault zones may affect the formation and accumulation of groundwater, hydrocarbon, ores and tectonically- and- structurally-controlled fluid flow in the brittle upper crust (e.g.,^1–3^). Fluids are of paramount importance since they control the effective stress during the seismic cycle, thus affecting fault mechanics and the overall style of deformation^4–7^. It has been demonstrated that both natural and human-induced earthquakes and seismic sequences can be triggered by fluid overpressure^7–13^. A detailed characterization of the fault architecture with direct constraints on the internal permeability structure of faults is, therefore, fundamental to (i) understand fault mechanics at all scales, (ii) develop refined models of fluid circulation in the brittle upper (seismogenic) crust and of the consequences thereof in terms of georesource formation and accumulation and (iii) mitigate the geological risk due to natural and induced earthquakes.

**Figure 1:**
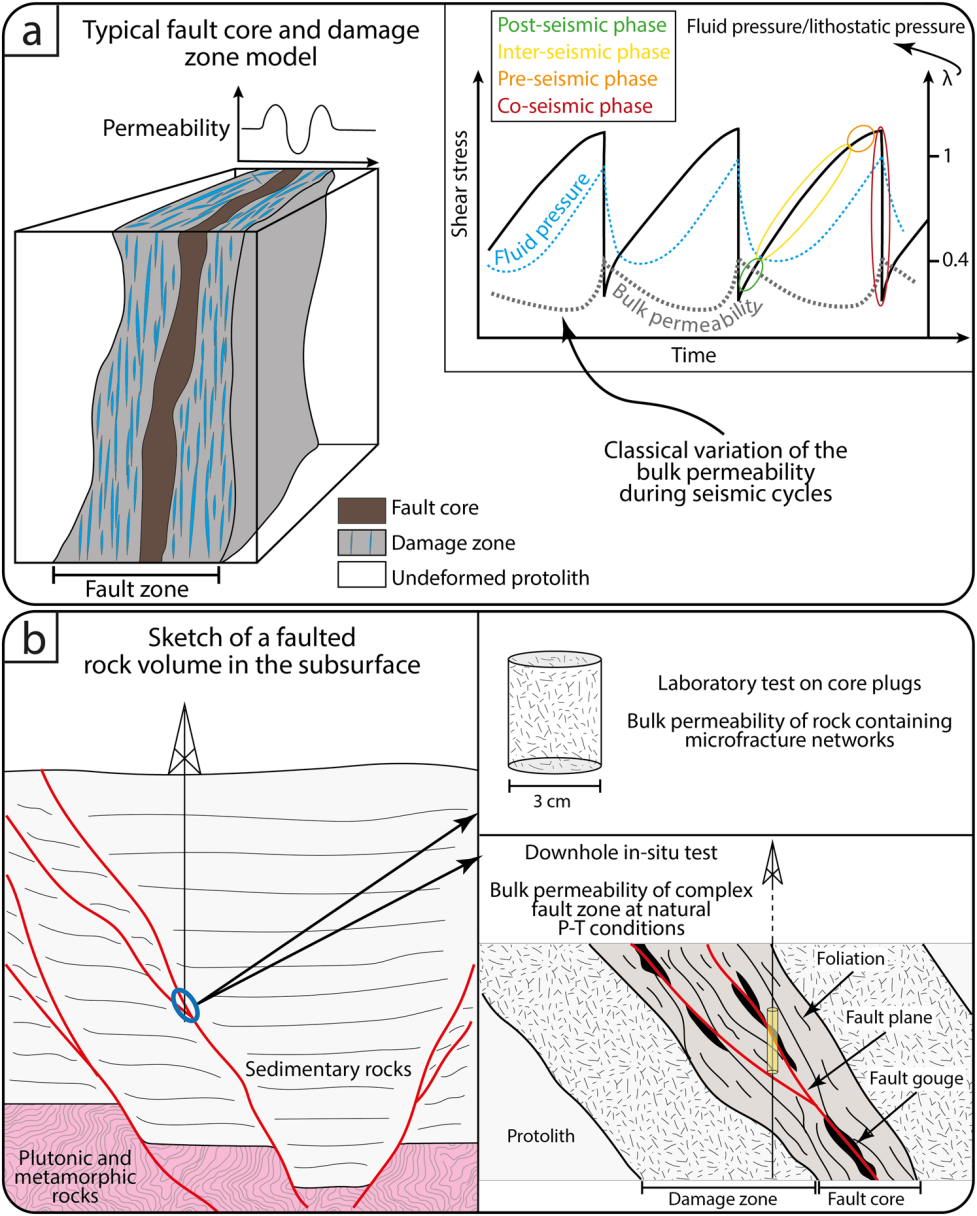
(**a**) Typical “fault core and damage zone” model of faults (redrawn and modified from^14^) in which low permeability fault cores provide an across-fault barrier to flow and high permeability and pervasively fractured dagame zones provide a preferential along-fault conduit. The classical variation of the bulk permeability during seismic cycles (i.e., through time) is also shown. (**b**) Sketch of the subsurface of a faulted volume and of the most adopted investigation methods for fault permeability determination. This figure has been created with Adobe Illustrator 2022 (https://www.adobe.com/products/illustrator.html).

In the typical “core and damage zone” model of faults, fault cores are depicted as providing across-fault barriers to flow, while pervasively fractured damage zones as along-fault conduits (e.g.,^14^; Fig. 1a). However, complex fault architectures may differ from this relatively simple paradigm as they contain secondary and fault-related structures associated with a distinct hydraulic behavior. The coexistence within heterogeneous fault architectures of structural domains with remarkably different hydraulic behaviors can cause bulk and local heterogeneities and anisotropies of the local permeability tensor. In detail, in addition to the primary permeability of the protolith (matrix permeability), the secondary structural permeability of a fault zone has been shown to be governed by the permeability of individual fault rocks, fractures, damage zone and by their 3D geometric architecture (e.g.,^6,15,16^). For example, fault cores are commonly rich in phyllosilicates, which, although typically extremely little permeable (e.g.,^17,18^), form effective hydrologic barriers only when they are continuous and physically interconnected. Open fractures and slip surfaces have an along-strike permeability governed by the distribution and connectivity of their apertures (e.g.,^19,20^). The presence of fault rocks characterized by planar tectonic fabrics (e.g., aggregates of bands of clay minerals and/or insoluble material) can also strongly affect the permeability within the rock volume leading, for instance, to remarkably different “across vs. along foliation” permeability structures, and thus significantly partitioning and modulating the circulation of fluids in the crust (e.g.,^6,19,21^). In addition, several studies have highlighted that faults are also characterized by anisotropic and complex hydraulic properties that vary through time (during an orogenesis or a seismic cycle; Fig. 1) in response to the development of different fault rocks (e.g.,^19,22,23^).

Recently, Ref.^24^ provided an updated compilation of existing fossil and active fault zone permeabilities from direct tests (e.g., downhole in-situ or laboratory analysis) and/or from rock porosity, image analysis, or subsurface fluid flow estimations. The comprehensive compilation highlights that the most adopted investigation methods are (i) laboratory analyses under controlled pressure–temperature (P–T) conditions on core plugs to calculate the bulk permeability of a few cm^3^ of rock containing microfracture networks and (ii) downhole in-situ tests to measure the bulk permeability of complex fault zones at natural P–T conditions (Fig. 1b). All this knowledge notwithstanding, a systematic and detailed characterization of fault permeability and variability within the framework of individual fault architectures is still missing.

Variably deformed structural domains can be preserved within fault systems and represent archives of the inherited geometric, kinematic, mechanic, and geochemical signature of a fault and its permeability evolution in time and space. These domains have recently been named “brittle structural facies—BSF” and defined as “deformed volumes of rock characterized by a given fault rock type, texture, color, composition, and age of formation” and “generally exhibiting sharp boundaries and complex crosscutting relationships with neighboring domains and whose unravelling is crucial to establish a relative temporal sequence of (de)formation” (^25^). The detailed identification of BSFs, their spatial distribution within fault zones, and the systematic analysis of their permeability properties offer the possibility to further advance our understanding of the (i) permeability structure of complex fault zones in 3D, (ii) hydraulic compartmentalization within the faulted crust, and possibly, (iii) variations thereof through space and time.

Aiming at further developing our understanding of the evolution of the permeability structure of faults, we report the first in-situ outcrop permeability values from two selected regional faults in the Northern Apennines of Italy representing outstanding examples of complex fault architectures. Our aim is to constrain the bulk permeability values of their BSFs so as to describe the permeability variability along and across those complex fault zones. We discuss the Zuccale and Boccheggiano faults (hereafter referred to as ZF and BF, respectively), which represent ideal sites to investigate heterogenous permeability properties due to their complex internal architecture and excellent exposure.

We show that the heterogeneous hydraulic properties of complex and long-lived faults drive the occurrence of (transient) low permeability volumes that can potentially generate fluid overpressure and cause seismogenesis. Fault permeability variations can be (i) remarkable (up to four orders of magnitude) in space, even within one single fault zone, and in time, during the fault continuous evolution, and (ii) generally associated with the development of BSFs that, each with its distinct hydraulic properties, form progressively and evolve during fault growth and repeated seismic cycles.

Our results represent a fist-order constraint on the present-day permeability at surficial conditions and, therefore, cannot be directly extrapolated to depth and back in time. To that end, it would be necessary to also consider the effects of P–T changes and different deformation conditions. Despite these limitations, this study represents a first step toward the in-situ outcrop detailed characterization of the permeability structure of complex faults.

## Geological background

**Figure 2:**
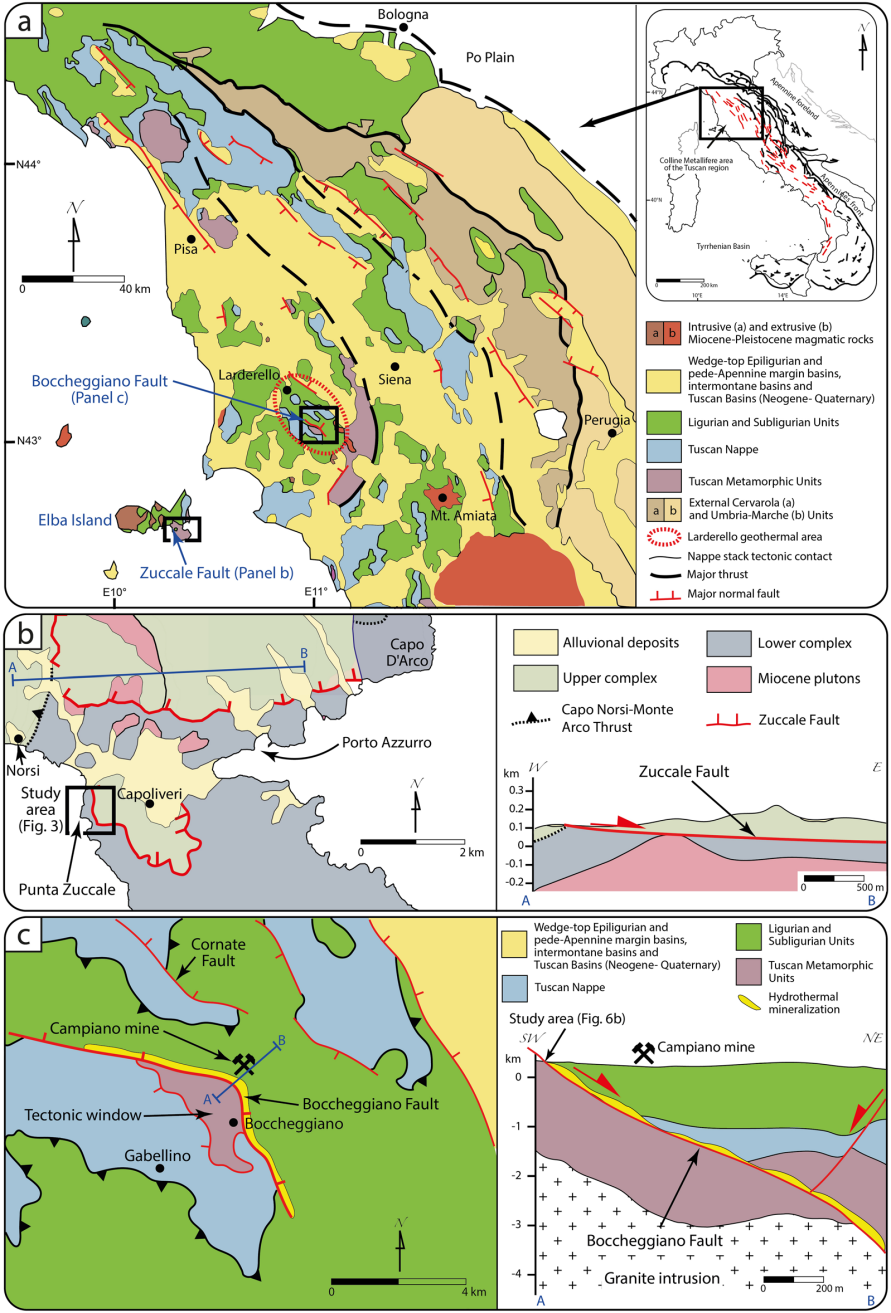
(**a**) Simplified geological map of the Northern Apennines (Italy) showing the tectonic units, the main thrusts and normal faults, and the location of the Zuccale and Boccheggiano faults. Redrawn and modified from^35^. (**b**) Simplified geological map of the study area (Punta Zuccale, Elba Island), where the Zuccale Fault is exposed. A cross section along the Zuccale Fault (modified and redrawn from^33^) is also shown. (**c**) Simplified geological map and cross section of the Boccheggiano Fault (redrawn and modified from^37^). This figure has been edited with Adobe Illustrator 2022 (https://www.adobe.com/products/illustrator.html).

The Zuccale and Boccheggiano faults are regional structures of the Northern Apennines of Italy (Fig. 2). The Apennines resulted from the Cenozoic convergence between Europe and Africa‐Adria plates during the W‐directed subduction of the Liguro‐Piemontese Ocean and of the Adria continental margin beneath Europe (e.g.,^26^). The first‐order structure of the Apennines results from the superposition of crustal shortening and extension associated with Tyrrhenian back‐arc development (e.g.,^26^). In the Northern Apennines shortening began during the late Oligocene leading to an eastward‐verging fold‐and‐thrust belt (e.g.,^26^; Fig. 2a). Rocks involved in the Northern Apennines consist of metamorphic Permo-Triassic siliciclastic (Verrucano Fm.) and Paleozoic sequences (Tuscan Metamorphic Units), ophiolite‐bearing metamorphic and non‐metamorphic complexes (Ligurian and Subligurian Units), and Mesozoic-Cenozoic marine carbonates (Tuscan Nappe; e.g.,^27^; Fig. 2a). Synorogenic siliciclastic flysches were progressively incorporated in the advancing fold‐and‐thrust belt (e.g.,^27^; Fig. 2a).

Post-orogenic extension in the Tyrrhenian sector of the Apennines began in the middle‐late Miocene and was associated with normal faulting, exhumation of subducted complexes, magmatism and structurally-controlled hydrothermalism and metallogenesis (e.g.,^28,29^; Fig. 2a). Extension progressively migrated toward the east and is currently active in the axial portion of the belt where locally overpressured fluids promote seismicity along (mainly) NW–SE striking normal fault systems (e.g.,^30,31^).

The low-angle Zuccale Fault (ZF) is located on eastern Elba (Tuscan Archipelago, inner Northern Apennines; Fig. 2a,b), which is made of a stack of variably metamorphosed and non-metamorphic tectonic slivers of the Tuscan Nappe and Ligurian Unit imbricated toward the NE (e.g.,^32^; Fig. 2b). The nappe stack hosts late Miocene monzogranitic intrusions (Monte Capanne and Porto Azzurro plutons, e.g.,^28^; Fig. 2a,b). The ZF discordantly cuts across the Calanchiole Shear Zone (CSZ), a top-to-E (compressional) shear zone defined by calc-mylonitic marble (e.g.,^33^).

The role and significance of the ZF within the framework of the Northern Apennines is still debated. It was initially interpreted as a LANF (low angle normal fault) accommodating late Miocene and Pliocene extension (e.g.,^34^) and this interpretation is still valid to many researchers. Subsequently, it has been suggested that the ZF represents the flat segment of an Aquitanian thrust reactivated during early Pliocene out-of-sequence thrusting (e.g.,^33,35^). Regardless of this debate and the regional impact of the ZF upon the local tectonic evolution, the ZF stands out as a remarkable and complex fault accommodating a kilometric displacement (Fig. 2b).

The Boccheggiano Fault (BF) is located in the Boccheggiano-Montieri area of the Northern Apennines (Fig. 2a,c) and belongs to the NW–SE striking and E-dipping extensional fault system controlling the Pliocene–Pleistocene structural architecture of the southern branch of the Larderello-Travale geothermal area (^36–38^; Fig. 2a). It represents the eastern tectonic boundary of the Boccheggiano tectonic window, where low-grade metamorphic sequences of the Tuscan Metamorphic Units crop out (^36^; Fig. 2c). We studied the BF as it offers the possibility to investigate the permeability structure of a complex fault that controlled (i) the exhumation of a metamorphic basement in the footwall of a normal fault and (ii) the hydrothermal outflow and associated ore deposit formation. Indeed, the BF cuts across at c. 3 km depth granitic intrusions (Fig. 2c) that represent the source of fluids responsible for polymetallic sulfide ore deposition within the damage zone of the BF, as shown by the interpretation of seismic reflection images^36^.

## Methods

Structural analysis and in-situ outcrop permeability measurements were carried out on the main structural elements of the ZF and BF (principal slip surface—PSS, brittle structural facies—BSF, and, where possible, undeformed host rock). In-situ outcrop permeability measurements from the ZF followed the description of fault architecture and BSFs by Ref.^35^, who provide the most recent characterization of the ZF as a patchwork of at least six BSFs formed at different times during long-term fault activity. Measurements of in-situ outcrop permeability along the BF were conducted according to our own identification of BSFs done by expanding upon the available fault characterization by Refs.^37,38^. To characterize the intrinsic primary permeability properties of PSS, BSFs and host rock, we excluded secondary fractures from our in-situ outcrop measurements. Moreover, the possible effects of superficial alteration that could lead to spurious results were avoided by systematically cleaning the measuring sites and skipping clearly weathered rock exposures. In-situ outcrop permeability measurements were carried out on selected BSFs both parallel and perpendicular to the bedding S_0_ (when preserved) and/or tectonic foliation S_n_ so as to investigate the effect of planar (primary and/or secondary) anisotropies upon the permeability properties within the fault architecture. In-situ outcrop permeability data have been acquired with a New England Research TinyPerm-3 air-minipermeameter calibrated by the manufacturer against known standards. The TinyPerm allows for the field investigation of rock (bulk) permeability in the 10^–11^–10^–15^ m^2^ range within volumes of rock of 1–1.5 cm^3^, even though controlled laboratory tests have demonstrated its capability to measure values as low as 10^–17^ m^2^^39^. In the bulk-rock permeability mode, the air-minipermeameter directly yields an estimate of the permeability based on the outgoing air flow rate from the instrument built-in compression vessel. Permeability values obtained from air-minipermeametry need to be corrected and standardized to be comparable with permeability values obtained from laboratory tests on rock plugs or image analysis^39,40^. In detail, air–minipermeameters yield either larger permeability values (by a factor of ca. 1.7) when compared with results derived from image analysis quantifications^40^, or lower permeabilities (− 37%) than those obtained from small (< 10 cm) rock plugs used in laboratory tests^39^. Permeability results are described below, illustrated in Fig. 8 and listed in Table S1. Further details on the data acquisition and analysis are provided in the Supplementary material S1.

## Results

### The Zuccale Fault: structural framework and BSFs

**Figure 3:**
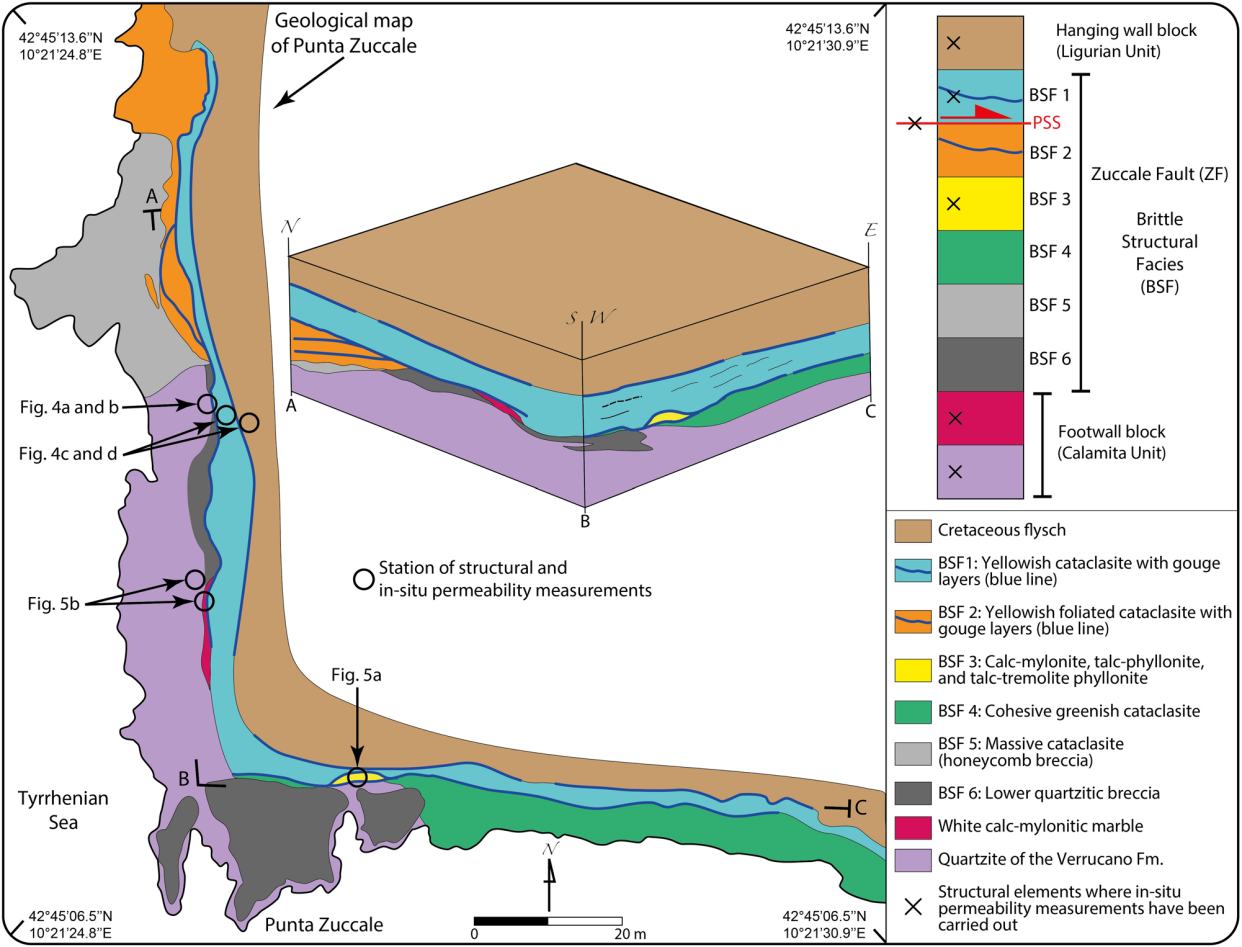
Geological map of Punta Zuccale showing (i) the distribution of the Brittle Structural Facies (BSF) of the Zuccale Fault as described in^35^ and (ii) the structural stations where in-situ outcrop permeability measurements were carried out. The block diagram illustrates the distribution of BSF along the NS and EW natural cross-sections that are directly accessible along the shoreline of Punta Zuccale (redrawn and modified from^35^ with Adobe Illustrator 2022 (https://www.adobe.com/products/illustrator.html).

**Figure 4:**
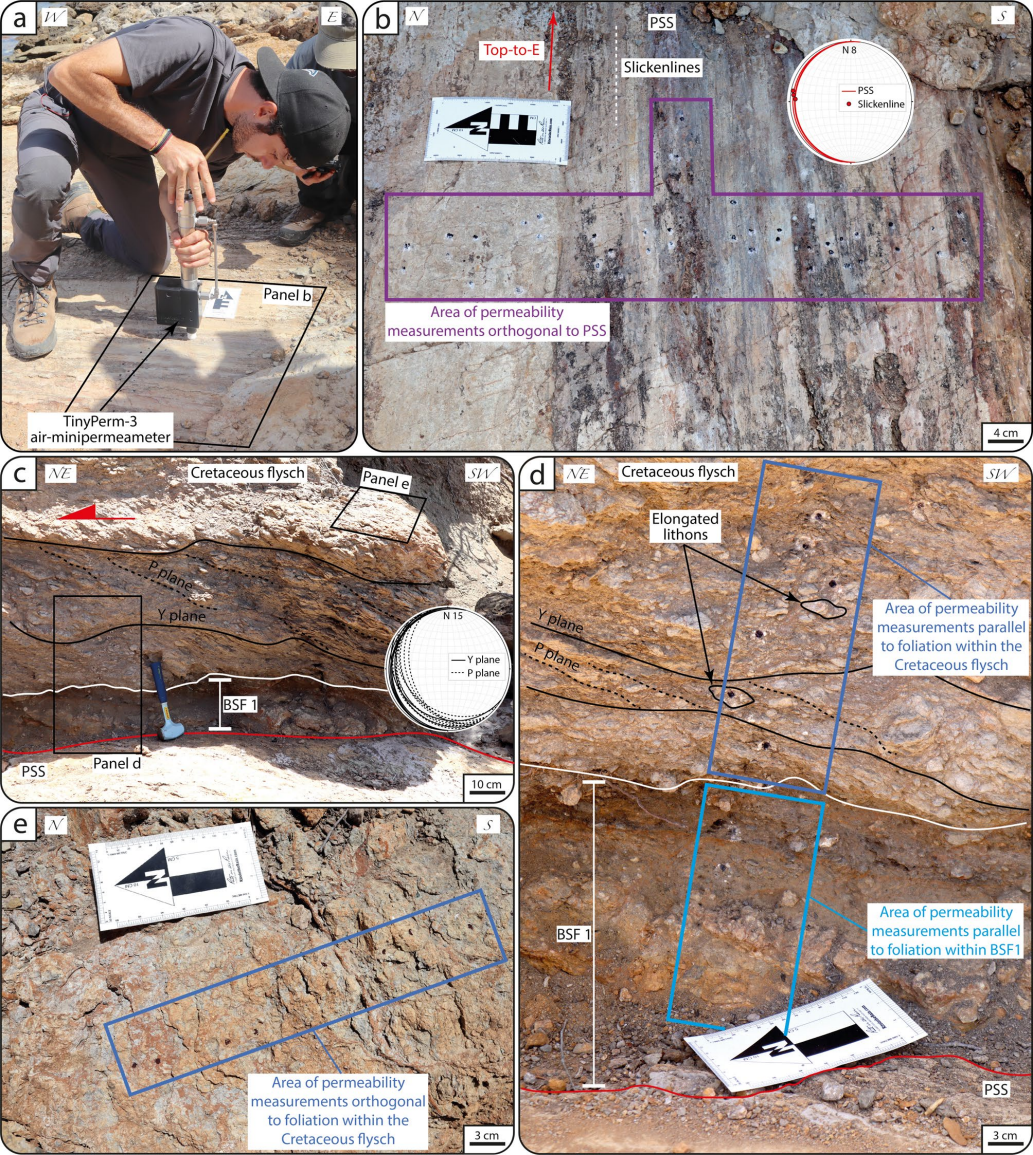
(**a**) In-situ outcrop permeability measurement by TinyPerm-3 air-minipermeametry orthogonal to the PSS of the ZF. (**b**) Striated PSS of the ZF accommodating top-to-E kinematics. The Schmidt net (lower hemisphere projection) plots the orientation of the PSS and of the relative slickenlines. (**c**) PSS, overlying BSF 1 and Cretaceous flysch containing Y-P tectonites the attitude of which is shown in the Schmidt net (lower hemisphere projection) in (**c**). (**d**) Detail of BSF 1 and Cretaceous flysch characterized by elongated lithons embedded within the foliated matrix. (**e**) Detail of the area of permeability measurements collected orthogonal to Y-P tectonites.

**Figure 5:**
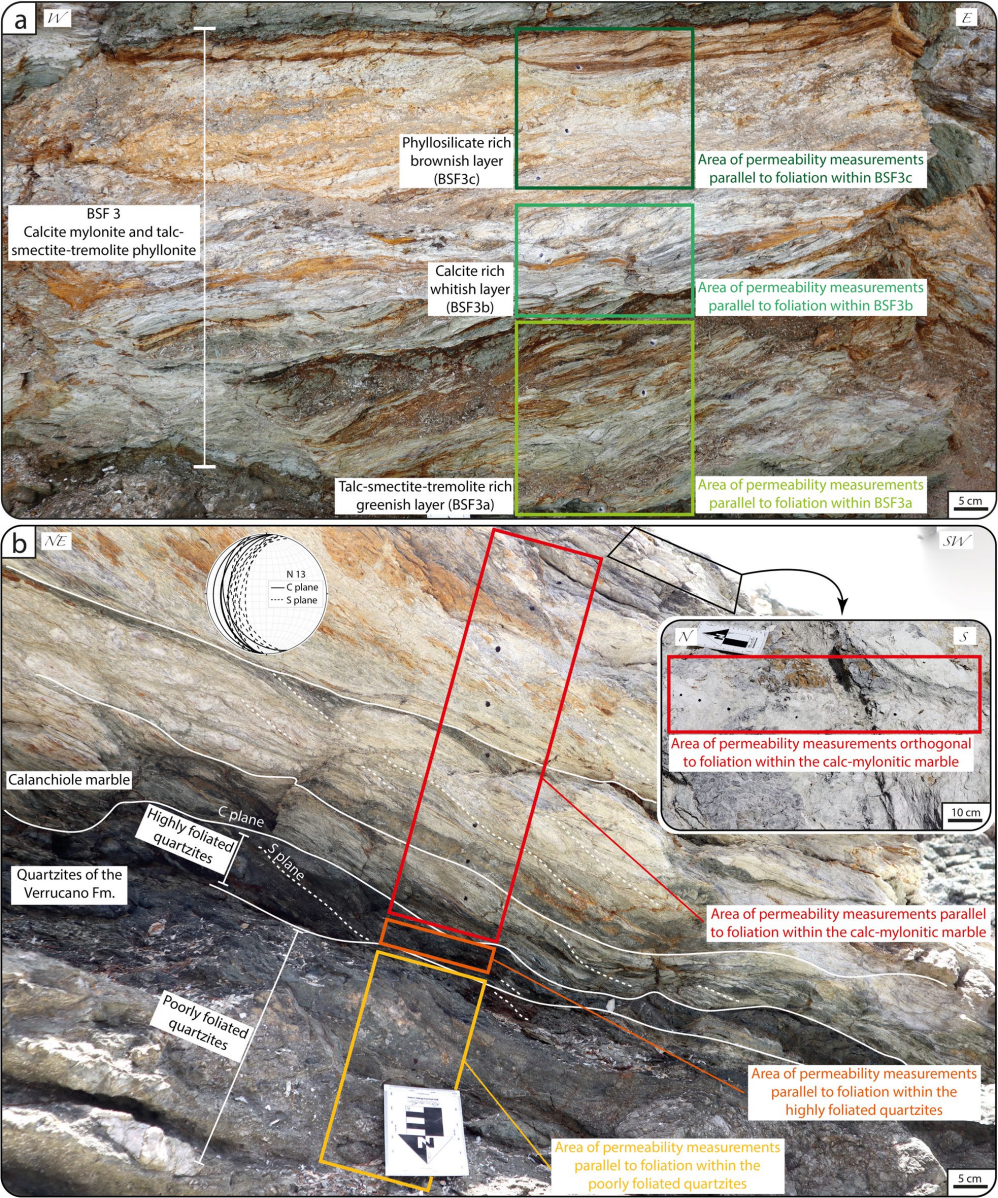
(**a**) BSF3 characterized by greenish (BSF3a), whitish (BSF3b), and brownish (BSF3c) layers. (**b**) Quartzites of the Verrucano Fm. and overlying Calanchiole marble. The quartzites are characterized by a lower poorly foliated portion and an upper highly foliated portion. The tectonic foliation within the Calanchiole marble is shown and plotted within the Schmidt net (lower hemisphere projection).

We studied the ZF at its best-known exposure, Punta Zuccale on Elba (Figs. 2b and 3), where continuous outcrops allow the detailed analysis of the fault internal architecture. We adopted the structural characterization of the ZF by Ref.^35^, which, from bottom to top of the Punta Zuccale section, distinguishes six BSFs (Fig. 3). We focused specifically on the following structural elements (Fig. 3), which are the most representative of the internal architecture of this mature fault:

- Principal Slip Surface (PSS): Discrete fault plane decorated by calcite slickenfibers indicating a top-to-E sense of shear (Fig. 4a,b).
- BSF 1: Yellowish foliated cataclasite with discrete gouge layers. The cataclasite matrix consists of secondary dolomite, calcite, quartz, clay minerals, and Fe-oxides with millimetric and centimetric clasts of limestone, quartzite, and granite. Gouge layers are indurated, greenish, cohesive, and matrix-supported and are locally foliated. BSF 1 rests above the PSS and is bounded by the Cretaceous flysch at the top (PSS; Fig. 4c–e).
- BSF 3: Calc-mylonite, talc-phyllonite, and talc-tremolite phyllonite lensoidal body. The phyllonite consists of a serrated alternance of well foliated greenish (BSF3a), whitish (BSF3b), and brownish (BSF3c) layers that are centimeter to tens of centimeter-thick and that reflect a compositional layering made up of talc-smectite-tremolite, calcite, and phyllosilicate rich layers (Fig. 5a).
- Cretaceous Flysch (hanging wall block): localized bands of foliated cataclasite embedding elongated sigmoidal lithons of less deformed to undeformed protolith (Fig. 4c–e).
- Quartzite of the Verrucano Fm. (footwall block): ca. 1 m thick cataclastic quartzite characterized by a poorly foliated basal portion and a highly foliated upper portion containing S-C tectonites indicating a top-to-E kinematics. Quartzites are bounded to the top by a calc-mylonitic marble (Fig. 5b).
- Calc-mylonitic marble (Calanchiole marble, footwall block): ca. 1 m thick tremolite and talc calc-mylonitic marble containing a pervasive S-C mylonitic fabric dipping ca. 40° to the west and indicating a top-to-E kinematics (Fig. 5b).

### The Boccheggiano Fault: structural framework and BSFs

**Figure 6:**
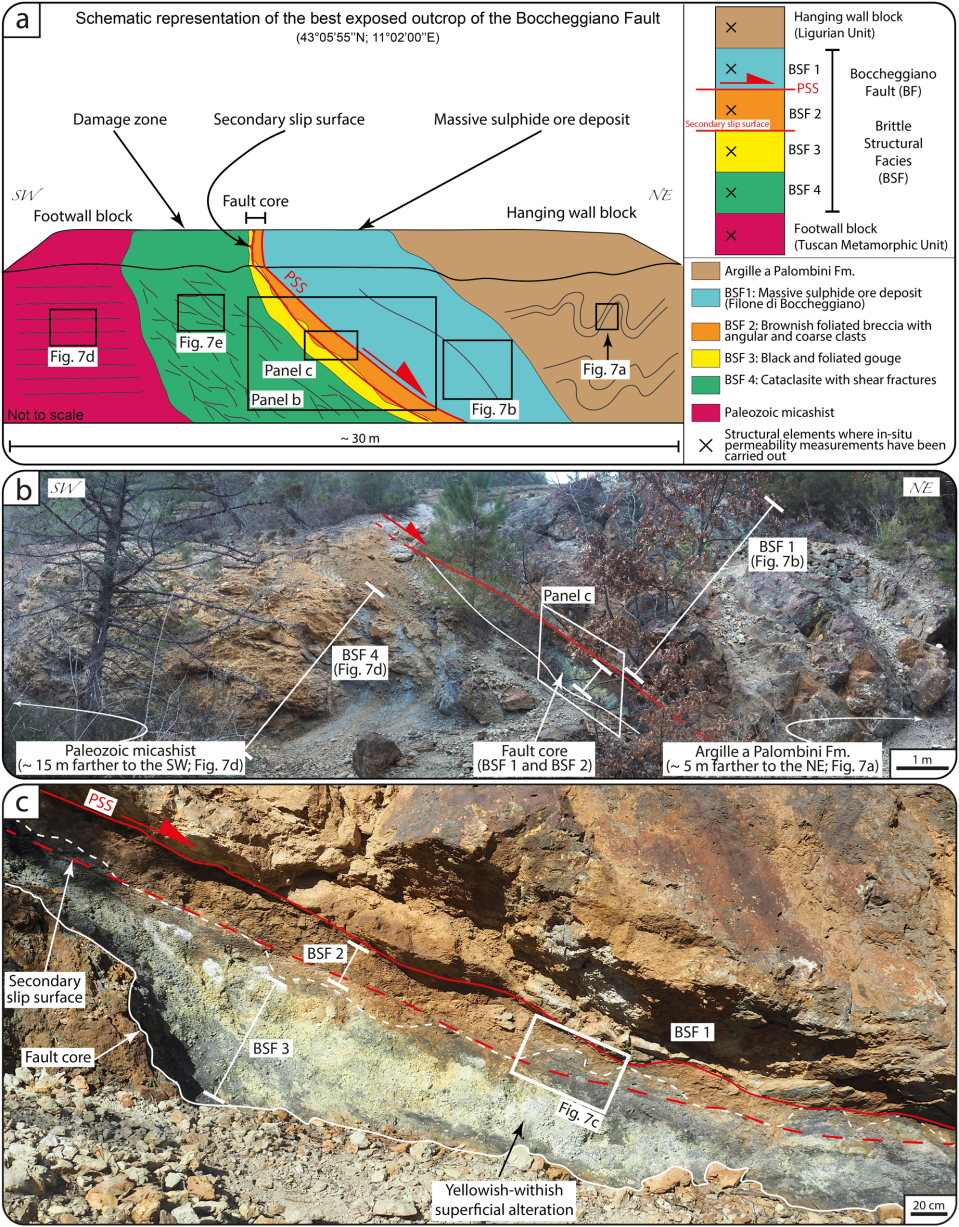
(**a**) Schematic representation of the best exposed outcrop of the Boccheggiano Fault where we carried out in-situ outcrop permeability measurements. The distribution of BSFs along the Boccheggiano fault is shown. (**b**) Outcrop of the Boccheggiano Fault showing the BSFs forming the complex architecture of the Boccheggiano Fault. (**c**) Detail of the BSF2 and BSF3 forming the fault core of the Boccheggiano Fault.

Our structural characterization of the BF expands upon Refs.^37,38^. In detail, fault rocks described by those authors are ascribed inhere to distinct BSFs (Fig. 6a).

**Figure 7:**
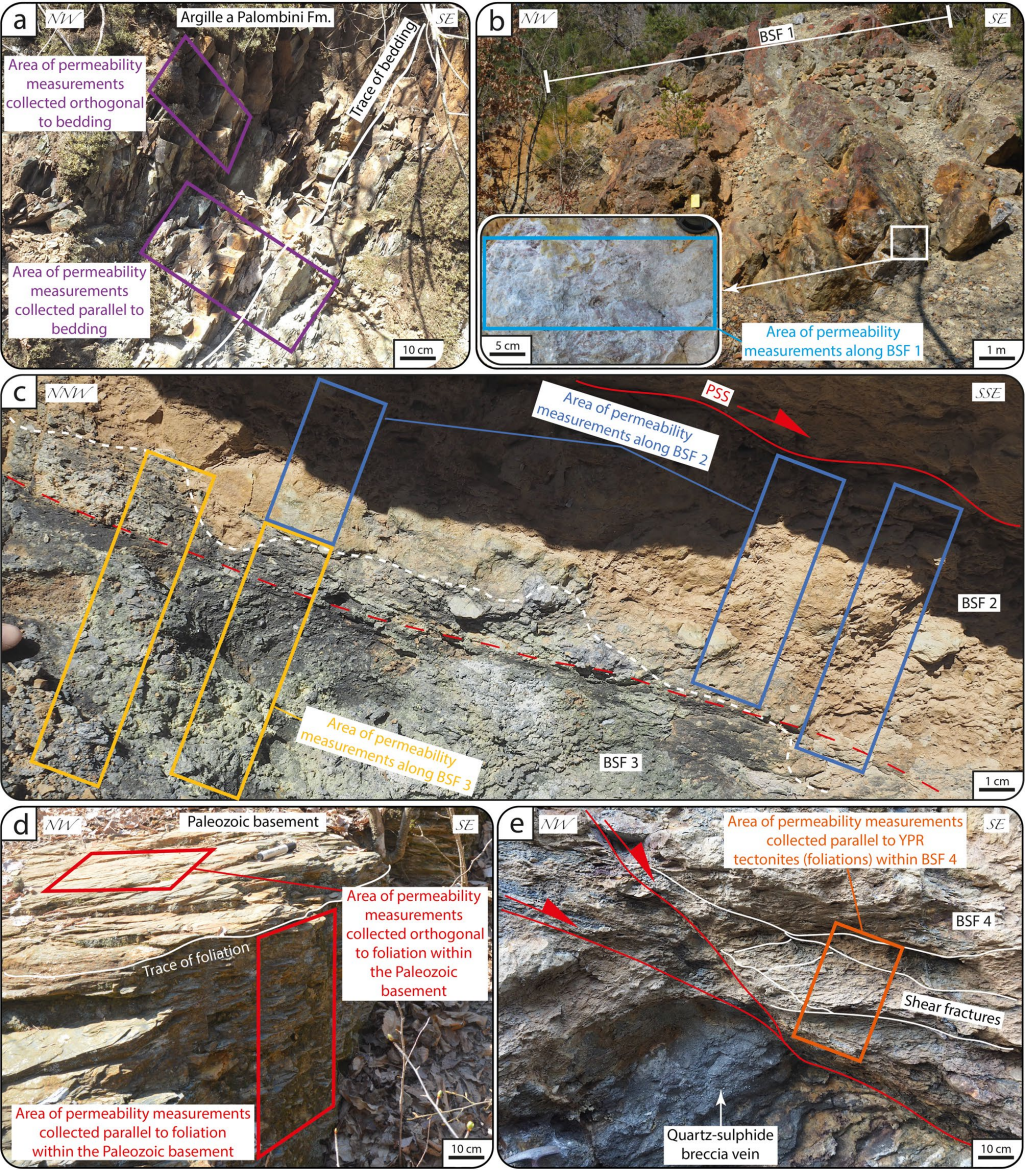
Stations of permeability measurements along the BF. (**a**) Shales belonging to the hanging wall unit (Argille a Palombini Fm.). (**b**) BSF1. (**c**) BSF2 (below the PSS) and BSF3. Note that the (irregular) boundary between BSF2 and BSF3 (white dotted line) is cut by a later secondary slip surface (red dotted line). (**d**) Micaschist of the footwall unit (Paleozoic basement). (**e**) BSF4 characterized by shear fractures.

The BF juxtaposes marine siliceous-carbonate rocks of the Cretaceous Argille a Palombini Fm. of the Ligurian Unit in the hanging wall against Paleozoic micaschist of the Tuscan Metamorphic Units in the footwall (^36–38^; Figs. 2c and 6a). The Argille a Palombini Fm. is well bedded, locally folded (Figs. 6a and 7a) and, in the immediate hanging wall of the BF, metasomatized and mineralized to form a massive sulfide ore deposit with quartz-adularia-sericite gangue minerals, with a maximum thickness ~ 10 m, and known as the “Filone di Boccheggiano” (which, hereinafter, we refer to as BSF 1; Figs. 6a,b and 7b;^37,38^).

The fault core is bounded above by a discrete PSS, has a maximum thickness of ~ 1 m, tapers up dip (Fig. 6a,b) and includes (i) a ca. 1 m-thick layer of brownish fault breccia with angular and coarse clasts of micaschist from the Paleozoic metamorphic basement and quartz-pyrite veins (BSF 2; Fig. 6a–c) and (ii) a black, ca. 50 cm-thick layer of cohesive foliated phyllosilicate-rick gouge containing millimetric sub-rounded clasts of micaschist of the Paleozoic metamorphic basement and quartz-pyrite veins (BSF 3;^37,38^; Fig. 6a–c). The boundary between BSF 2 and BSF 3 has an irregular shape and is cut by a secondary slip surface (Figs. 6c and 7c). Paleozoic micaschist in the footwall are characterized by a pervasive SL fabric that, within the first ~ 10 m from the fault surface, is overprinted by shear fractures defining the fabric of the ~ 10 m thick footwall cataclasite (BSF 4; Figs. 6a and 7d;^37,38^). The footwall cataclasite also contains (i) sub-horizontal quartz-sulfide breccia veins forming pinch and swell massive bodies and (ii) NE-dipping, synthetic normal faults (^37,38^; Fig. 7e).

### Permeability

The results of in-situ outcrop permeability measurements from the ZF and BF are first described separately but are then discussed together to allow for general conclusions on the permeability architecture of complex fault zones to be drawn.

#### Permeability structure of the Zuccale fault

**Figure 8:**
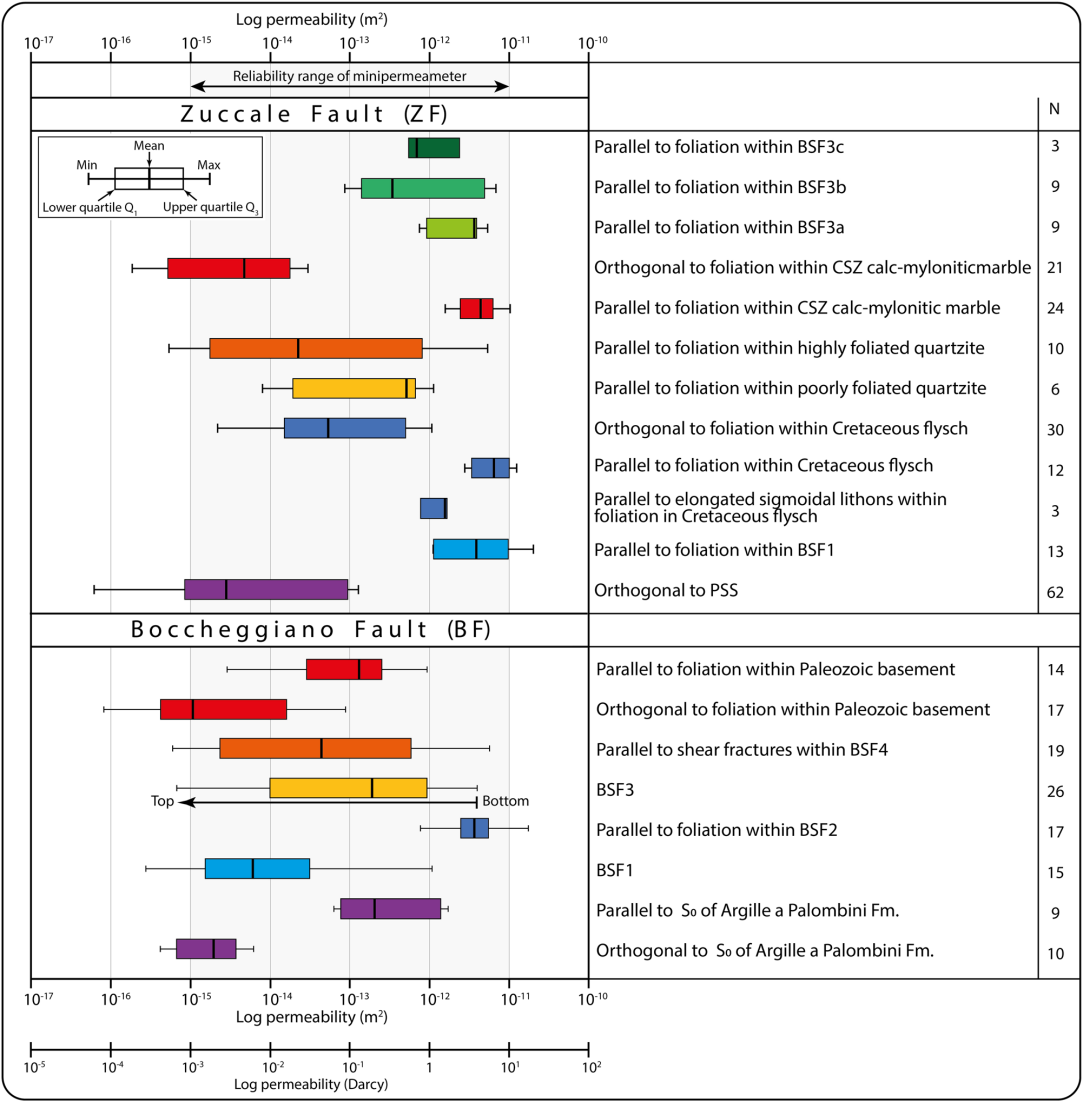
Results from in-situ outcrop air-permeability (m^2^, D) along the Zuccale Fault and Boccheggiano Fault. The corresponding BSF, structural elements, and orientation of measurements with respect to the tectonic foliations are reported. N: number of measurements. Each box of the box-and-whiskers plot represents the range between the 1st and 3rd quartile of the distribution. The whole data range is represented by the extension of the whiskers.

The PSS yielded the lowest mean permeability (~ 3 × 10^–15^ m^2^) of the entire ZF architecture, with values orthogonal to the PSS ranging between ~ 10^–17^ and ~ 10^–13^ m^2^ (Fig. 8). Permeability values parallel to the foliation planes within BSF 1 range between 10^–12^ and 10^–11^ m^2^, with a mean of ~ 4 × 10^–12^ m^2^ (Fig. 8).

Permeability orthogonal to the foliation within the Cretaceous flysch has a mean value of ~ 5 × 10^–14^ m^2^, ranges between ~ 10^–15^ and 10^–12^ m^2^, and is up to 3 orders of magnitude lower than the permeability measured parallel to the foliation, which ranges between ~ 5 × 10^–12^ and 10^–11^ m^2^ (Fig. 8). Values from parallel to the elongated sigmoidal clast embedded within the tectonites range between ~ 10^–12^ and 2 × 10^–12^ m^2^. Quartzites of the Verrucano Fm. yield permeability values that vary as a function of the intensity of foliation. In detail, the basal and poorly foliated portion has minimum and mean values up to one order of magnitude larger than those measured along the upper highly foliated portion (between ~ 8 × 10^–15^ and 10^–12^ m^2^ and mean of ~ 5 × 10^–13^ m^2^ in the former and between ~ 5 × 10^–16^ and 5 × 10^–12^ m^2^ and mean of ~ 6 × 10^–14^ m^2^ in the latter; Fig. 8). The permeability parallel to the mylonitic foliation within the CSZ calc-mylonitic marble is up to five orders of magnitude higher (10^–12^–10^–11^ m^2^) than that measured orthogonal to the mylonitic foliation (10^–16^ ÷ 10^–14^ m^2^; Fig. 8).

BSF 3 has permeability values between 10^–13^ and ~ 10^–11^ m^2^, although slightly different values have been obtained from the three different domains. In detail, the permeability of the BSF 3a, b, and c has mean values of ~ 4 × 10^–12^, ~ 3 × 10^–13^, and ~ 7 × 10^–13^ m^2^, respectively (Fig. 8).

#### Permeability structure of the Boccheggiano fault

The hanging wall unit (marine siliceous-carbonate rocks of the Argille a Palombini Fm.) has permeability values measured parallel to S_0_ that are from one to four orders of magnitude higher than those measured orthogonal to S_0_ (between ~ 4 × 10^–14^ and 2 × 10^–12^ m^2^ for the former and between ~ 4 × 10^–16^, and ~ 6 × 10^–15^ m^2^ for the latter; Fig. 8). BSF 1 has permeability values between ~ 3 × 10^–16^ m^2^ and 10^–12^ m^2^, with a mean value of ~ 6 × 10^–15^ m^2^ (Fig. 8). BSF 2 has the highest permeability measured along the BF, between 10^–12^ and 10^–11^ m^2^, with a mean value of 4 × 10^–12^ m^2^ (Fig. 8). The permeability of BSF 3 systematically decreases by up to 4 orders of magnitude (from ~ 4 × 10^–12^ m^2^ to ~ 7 × 10^–16^ m^2^; Fig. 8) toward the secondary slip surface (i.e., from bottom to top), which cuts the BSF 2–BSF 3 boundary (Fig. 8). The permeability parallel to shear fractures within BSF 4 ranges from ~ 5 × 10^–12^ to ~ 6 × 10^–16^ m^2^ (below the actual reliability limit of the air-minipermeameter) with a mean value of ~ 7 × 10^–14^ m^2^ (Fig. 8). Permeability within the footwall Paleozoic basement exhibits very different values depending on whether it is measured parallel or orthogonal to the foliation (Fig. 8). In detail, the permeability orthogonal to foliation is the lowest measured for the entire BF, as it is up to four orders of magnitude lower than that measured parallel to the foliation and has a minimum and mean value of ~ 8 × 10^–17^ m^2^ and ~ 1 × 10^–15^ m^2^, respectively (Fig. 8).

## Discussion

### Rationale

**Figure 9:**
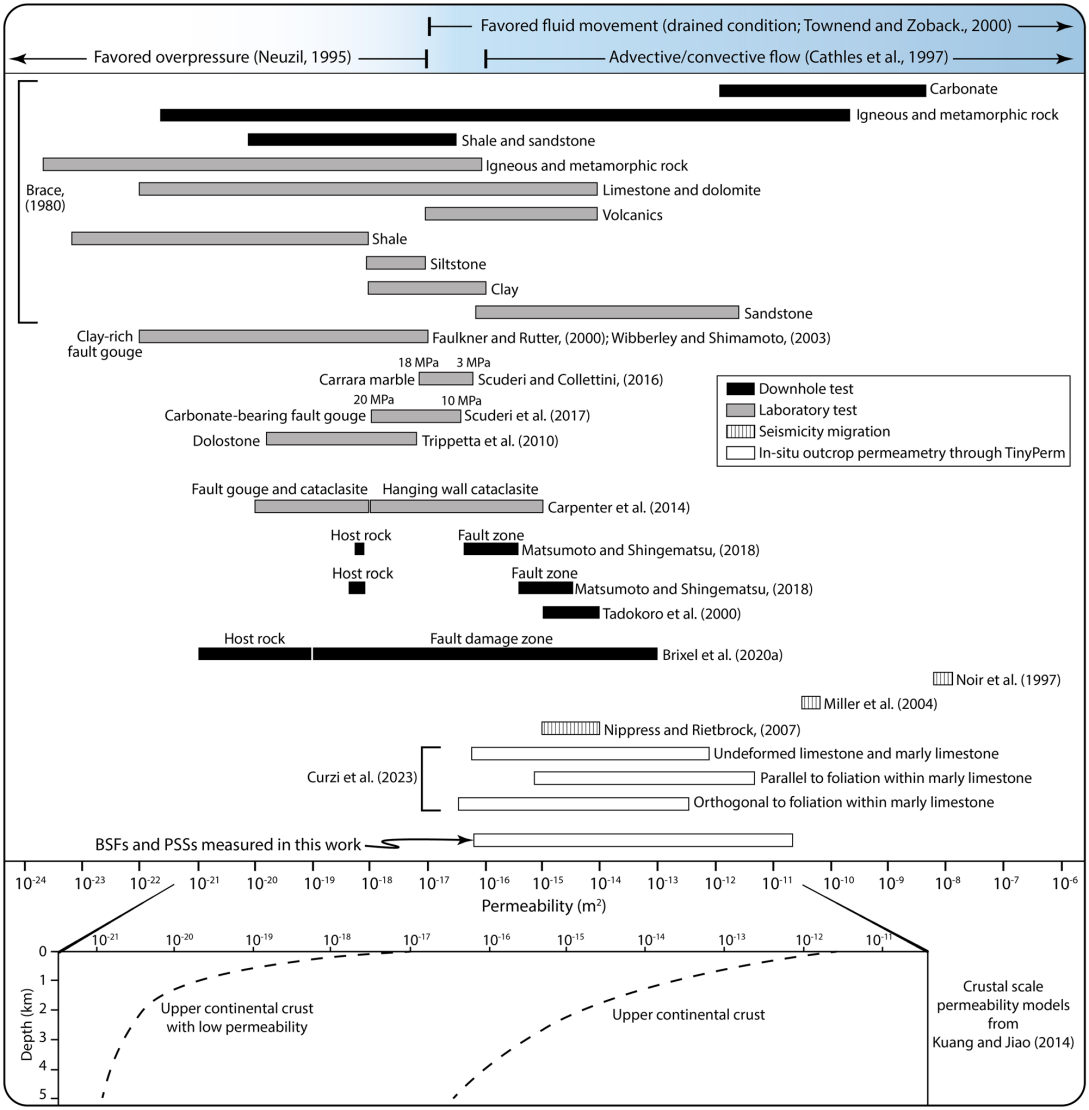
Permeability range for different rocks, fault rocks, and crustal volumes as constrained by downhole and laboratory tests, seismicity migration, and in-situ outcrop permeametry. Permeability values as predicted by upper crustal scale permeability models are also shown by the black dotted curves.

Our results constrain the present-day permeability of the studied fault rocks at the outcrop. Any extrapolation to depth (e.g., at seismogenic depth) would need to consider the effects of increasing confinement and temperature and the presence of hydrothermal fluids (e.g.,^37,38^), which generally promote an overall decrease of permeability (e.g.,^41^). Indeed, (i) laboratory permeability tests on different lithotypes (e.g., marble, carbonate and dolostone) and (ii) comparison of downhole and laboratory permeability tests document a general decrease of permeability with increasing confining pressure and/or depth (e.g.,^7,15,42–44^; Fig. 9). On this ground, we can use our results only to constrain bulk relative permeability differences between BSFs forming the complex internal architecture of the investigated fault zones. Also, as already mentioned, air–minipermeametry tends to yield (i) higher permeability values (by a factor of ca. 1.7) compared to results from image analysis quantifications^40^, but (ii) lower values (− 37%) than those from small (< 10 cm) rock plugs for laboratory tests^39^. Moreover, any comparison of gas (e.g., air) and water permeability data should consider the Klinkenberg effect^45^, which enhances gas flow (relative to water flow) due to slip on pore walls, with the net result that gas permeability is generally higher than water permeability. This notwithstanding, it has been shown that the Klinkenberg effect-related difference between gas and water permeability ranges between 4 and 16% for permeability values < 10^–18^ m^2^, that is, lower than those obtained in this work^46,47^. Hence, our air permeability values can be used and interpreted as representative of also water permeability.

As a whole, the most adopted investigation methods to constrain permeability, including downhole and laboratory tests and natural or induced seismicity migration^7,9,15,42–44,48–55^, highlight that the permeability of the (faulted) upper brittle crust spans more than 15 orders of magnitude, ranging from drained conditions (> 10^–17^ m^2^) to favored overpressure conditions (< 10^–17^ m^2^;^56–58^; Fig. 9). Moreover, crustal-scale permeability models show that the permeability greatly varies as a function of site-specific, local and regional conditions (e.g., stress, lithotype and structural damages;^59^; Fig. 9). In this context, in contrast to other commonly adopted investigation methods (Fig. 9, Table 1), in-situ outcrop permeability analysis along continuously exposed fault zones offers the remarkable advantage to investigate and reconstruct the hydraulic properties of complex faults composed of multiple, heterogeneous, and discontinuous structural elements (e.g., PSS, BSFs) possibly even formed at different times during faulting. We stress, therefore, that in-situ outcrop permeability measurements from exhumed fossil faults and fault zones only represent a first step toward (i) bridging the gap between different investigation methods, (ii) strengthening the knowledge about relationships between complex fault-related structures and permeability, and (iii) investigating the 3D permeability structure of laterally discontinuous fault zones and their associated BSFs.

**Table 1:** Summary of the most adopted methods to directly/indirectly constrain fault rock permeability (summarized from^24^).

	Method	P–T conditions representative of the measured permeability	Scale of the investigated hydraulic/structural domain	Measured element	Temporal constraints on the analyzed fault rock
Indirect investigation methods	Image analysis of microfracture and/or pore geometry	Ambient	Microscale	Porosity	Relative time constraints from sampled rocks
	Cross-borehole tests	Environmental (non-surficial) condition	Seismic scale	Bulk	Relative time constraints based on seismic reflection profiles or sampled core plugs
	Injection test	Environmental (non-surficial) condition	Seismic scale	Bulk	Relative time constraints based on seismic reflection profiles or sampled core plugs
Direct investigation methods	Core plug laboratory test	Environmental (non-surficial) condition (up to instrumental limits)	Microscale	Matrix and porosity	Relative and/or absolute time constraints from sampled rocks and forming minerals
	In-situ outcrop permeametry of exhumed faults and fault zones	Ambient	Scale of the exposed fault and fault rocks (outcrop scale, mesoscale, centimeter scale)	Bulk for each juxtaposed BSF	Relative time constraints (based on cutting relationships observed in the field) and possible absolute time constraints (based on radiometric dating of fault rocks) of each analyzed fault rock

### Permeability of complex faults

The permeability of the complex architecture of the studied faults ranges between ~ 10^–17^ and 10^–11^ m^2^ (Fig. 8), thus documenting remarkable variations of their hydraulic properties. BSFs identified within the studied faults are characterized by (i) limited lateral continuity due to their wedge/lensoidal shape, (ii) different mineralogy and/or degree of cementation, and (iii) different spacing and intensity of internal fabric, all of which control and compartmentalize the deformed rock volume^6,34,35,37,38,60^. Indeed, our observations document that:

(i) the highest values of permeability are from poorly/moderately indurated cataclasite and fault breccia (BSF1 and Cretaceous flysch of ZF and BSF2 of BF; Figs. 4c,d, 6c, and 8), where little fault rock cementation accounts for high porosity and permeability;
(ii) the lensoidal BSF3 along the ZF is characterized by an internal permeability that, as a function of mineralogy, varies from ~ 4 × 10^–12^ m^2^ of the talc-smectite-tremolite rich layers (BSF3a) to ~ 3 × 10^–13^ m^2^ for the phyllosilicate rich layers (BSF3c), and ~ 7 × 10^–13^ m^2^ for the calcite rich layers (BSF3b; Figs. 5a and 8);
(iii) the lower the spacing of foliation, the lower the permeability, as shown by quartzites of the Verrucano Fm. along the ZF (Figs. 5b and 8).

The most efficient hydraulic barriers for sub-vertical fluid flow are:

(i) primary anisotropy planes (S_0_, when preserved) as shown by the Argille a Palombini Fm. in the hanging wall of the BF (mean permeability values in the order of 10^–15^ m^2^; Figs. 7a and 8);
(ii) the PSS that has minimum and mean values of ~ 10^–17^ and ~ 10^–15^ m^2^, respectively (Fig. 8), as documented along the ZF (Fig. 4a,b);
(iii) secondary (tectonic) foliation planes that are systematically characterized by permeability values measured orthogonally to the foliation from three to five orders of magnitude lower than those measured parallel to the foliation (Fig. 8), as documented within (1) the flysch (where orthogonal and parallel values ranges from 10^–15^ to 10^–11^ m^2^, respectively) and mylonitic marble (with orthogonal and parallel values ranging from 10^–16^ to 10^–11^ m^2^, respectively) along the ZF (Figs. 4c–e and 5b) and (2) the Paleozoic basement (with orthogonal and parallel values ranging from 10^–17^ to 10^–13^ m^2^, respectively) in the footwall of BF (Fig. 7d).

The very low permeability (down to 10^–17^ m^2^) orthogonal to the PSS and the primary and secondary foliation (Fig. 8) shows that these structural elements form effective hydraulic barriers to significant across fluid flow. Hence, depending on the dip angle of fault zones and foliation, these structural elements can represent hydraulic barriers in the sub-vertical or sub-horizontal dimension, causing effective compartmentalization of rock volumes. Results from the Cretaceous flysch along the ZF also highlight that the permeability parallel to the foliation is higher than that measured within the elongated centimetric sigmoidal lithons embedded within the foliation (Figs. 4d and 8). Hence, rock volumes characterized by lithons embedded within foliation planes can generate discontinuous hydraulic properties along the lateral dimension, thus promoting parallel-to-foliation fluid flow, which is instead locally hindered by poorly (or not at all) deformed lithons.

As recently documented, BSFs may differ in terms of their absolute age of formation as they progressively develop during potentially long-lived faulting^25,35^. Thus, the hydraulic properties of any fault zone may vary both in space (e.g., depending on the lateral continuity of BSFs and their internal mineralogical/structural heterogeneities) and time (e.g., depending on fault rejuvenation due to the development of newer BSFs). As observed along the BF, its BSF3 is characterized by a decrease of permeability toward the well-developed secondary slip surface (i.e., from bottom to top; Fig. 8), which cuts the BSF2-3 boundary (Figs. 6c and 7c). Hence, we propose that repeated slip along secondary slip surfaces induced pore compaction and possibly sealing causing a bulk permeability decrease within the adjacent portions of the BSF. This suggests that the evolution of permeability through time can be strongly affected by the development of younger and interdigitated BSFs (with irregular shapes, different structural features and variable mineralogy) and by subsequent deformation localization. In other words, our data suggest that long-lasting faulting continuously and repeatedly modifies and compartmentalizes the hydraulic properties of faults, which therefore transiently steer the development of volumes prone to act as hydraulic conduits and/or barriers in the brittle upper crust.

### Implications on fluid flow and seismicity

Natural and induced seismicity are often related to fluid overpressure, which is indeed considered one of the primary mechanisms facilitating seismic slip (e.g.,^4,7,8,11^). According to the well-established fault-valve behavior and to recent geochemical, hydrogeochemical, and seismological constraints^4,6,9,12,31,61^, fluid pressure in fault zones commonly increases during the inter-and pre-seismic phases causing instability and rupturing. During repeated seismic cycles, the localization of seismic ruptures is also controlled by local perturbations of the stress field and strain rate, frictional resistance, and amount and distribution of weak phases (e.g.,^60,62,63^).

**Figure 10:**
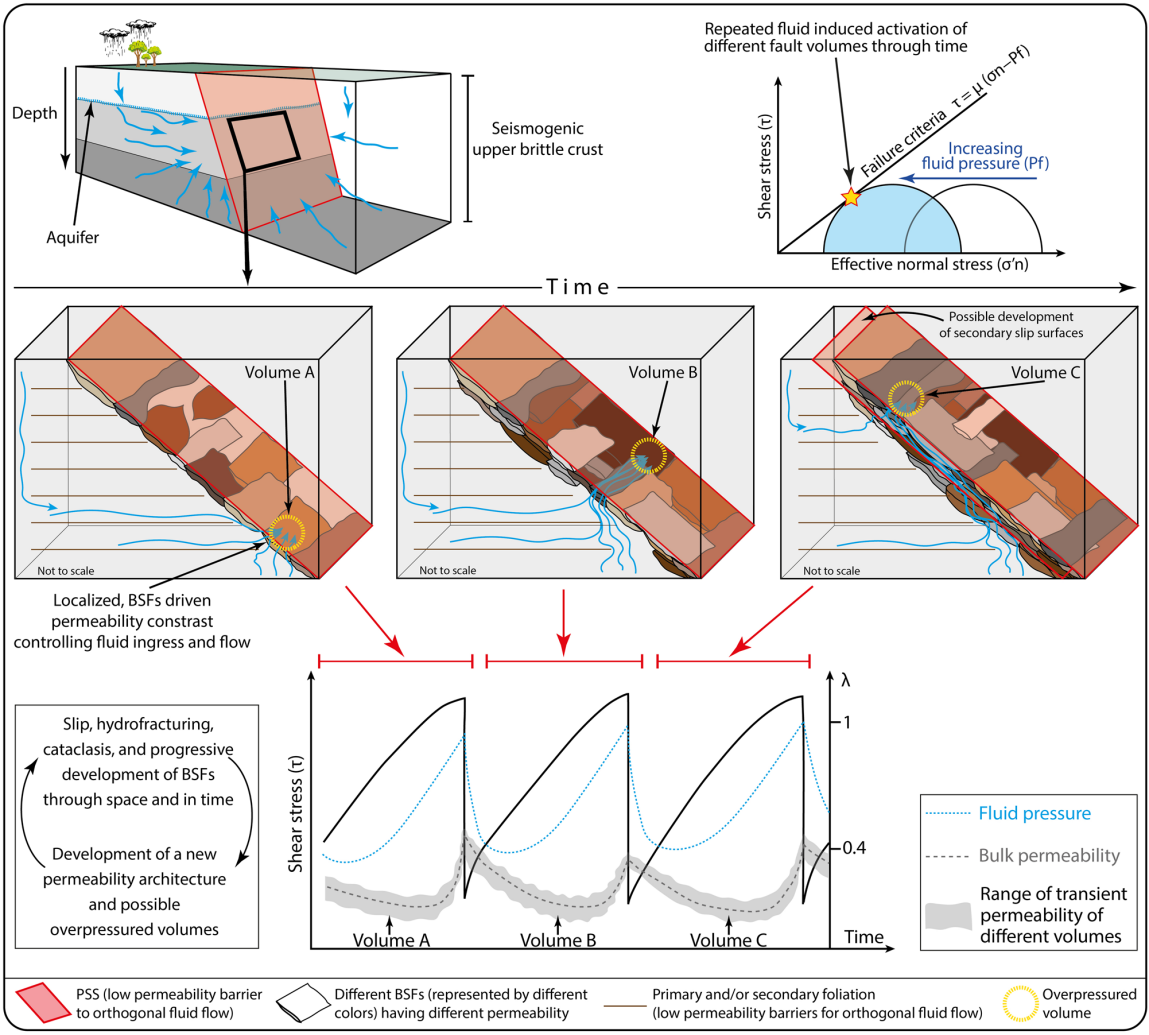
During progressive deformation, BSFs (and possibly secondary slip surfaces) can gradually develop leading to a continuously evolving permeability architecture in a fault zone with localized permeability contrasts enhancing fluid ingress and flow. In the case of development of overpressured volumes and seismic slip can be promoted. This process can be reiterated during the entire life span of faults. The range of the bulk permeability and the transient variation of the BSF-driven permeability during seismic cycles (i.e., through time) is also shown. This Figure has been created with Adobe Illustrator 2022 (https://www.adobe.com/products/illustrator.html).

We propose a model wherein the spatial and temporal variations of hydraulic properties associated with evolving complex fault architectures also play a role in localizing seismic rupturing (Fig. 10). In detail, the transient low permeability of specific BSFs can promote transient fluid accumulation, ponding, and overpressuring in different volumes of the fault architecture, eventually causing hydrofracturing, mechanical instability and earthquake nucleation (Fig. 10). As documented from the Apennines and elsewhere (e.g., California, Taiwan, and Japan; e.g.,^9,30,64–66^), dilatant processes take place during co-seismic rupturing and fluid pressure rapidly drops. At the same time, though, progressive faulting can promote a continuously evolving permeability structure characterized by low permeability volumes (Fig. 10;^67,68^). Therefore, new (i) pathways for fluid ingress and flow and (ii) low permeability volumes can favor fluid accumulation and overpressuring (inter- and pre-seismic phase), thus promoting seismic ruptures (co-seismic phase) in different volumes of the same fault (Fig. 10). By upscaling the proposed model to the brittle upper crust and integrating it through time (by considering repeated seismic cycles), we envisage a dynamic and transient and oscillating permeability structure, characterized by cyclic and short-lived changes of hydraulic properties in response to the deformation-related development of BSFs (Fig. 10).

Many studies have provided estimations of subsurface permeability through direct and (in particular) indirect analyses, including (i) downhole measurements, (ii) migrating patterns of induced and/or natural seismicity, (iii) groundwater temperature measurements of tectonically active areas, and (iv) CO_2_ leakage along faults^9,13,24,51,69,70^ (Fig. 9). Among those, some have also shown that the permeability along buried fault zones is transient and changes through space in response to the development of fracture corridors within damage zones, likely associated with co-seismic ruptures^23,70–72^. In detail, it has been proposed that the transient character of fault permeability is strictly controlled by the seismic cycle, with an up to 14-fold decrease of bulk permeability (up to high crustal permeability values of > 10^–10^ m^2^;^9,54^) during pre- to co-seismic dilatancy and fracture corridors, and post- to inter- seismic recovery of fault permeability due to fracture sealing and development of clay-rich gouges^13,67,73^. On this ground, the data presented in this work (i) help provide direct in-situ outcrop permeability constraints on complex fault architectures and (ii) highlight that fault-related permeability recovery and variation in space and time are genetically associated with fault BSFs and PSSs. In this context, our data offer a step toward 4D models of deformed crustal zones, which commonly (i) provide the static bulk permeability structure of deformed zones and (ii) highlight the spatial variation of permeability within exhumed deformed rock volumes (e.g.,^74–76^). Indeed, the high accuracy of such models notwithstanding, a dynamic conceptualization of the permeability structure associated with long-term fault-related deformations (i.e., transient variation of permeability through space and in time) is often neglected. In other words, our approach can be adopted to (i) provide further constraints on dynamic models of crustal permeability highlighting the transient nature of fault-related permeability, (ii) improve the knowledge of fault-related crustal permeability, and therefore (iii) allow an even more detailed reconstruction of fluid pathways and overpressuring along deformed (possibly tectonically active) crustal zones. In this context, seismic monitoring through high resolution data such as Vp/Vs anomalies, electrical conductivity and hydraulic pressure at depth (as in the Apennines; e.g.,^9,30,31,77^), can lead to the advanced understanding of complex faulting histories, with significant bearings on seismic risk mitigation.

## Conclusions

Fault zones are commonly characterized by complex architectures which can be described by the juxtaposition of distinct BSFs. Here we show that BSFs may be characterized by very different hydraulic properties that (i) change through space and in time and (ii) define rock volumes that are more prone to seismic slip and/or fluid flow. Our results shed further light on the complex, transient, and dynamic behavior of faults in the brittle upper crust. We believe that an even more detailed characterization of mechanical, petrophysical, and geochemical properties of BSFs may offer key insights in the mitigation of the geological risk associated with natural and induced earthquakes.

## Supplementary Information

Supplementary Information.

## Data Availability

Analytical data are available in Supporting Information S1.
