# Supplementary material for: Constraints on upper crustal fluid circulation and seismogenesis from in-situ outcrop quantification of complex fault zone permeability
Source: Sci Rep. 2023 Apr 5;13:5548. doi: 10.1038/s41598-023-32749-4 (PMC10076323; doi:10.1038/s41598-023-32749-4)
Supplement: Supplementary file 1 — Supplementary Information. [file 41598_2023_32749_MOESM1_ESM.docx]

**Supplementary Material**

**In-situ outcrop quantification of permeability properties of complex fault zones: new perspectives on fluid circulation and seismogenesis in the upper crust**

M. Curzi^1*^, F. Giuntoli^1^, G. Vignaroli^1^, G. Viola^1^

1. Dipartimento di Scienze Biologiche, Geologiche ed Ambientali – BiGeA, Università degli studi di Bologna, Via Zamboni 67, Bologna, 40126, Italy

Content of this file:

Methods and Table S1.

The TinyPerm is a portable handheld air permeameter used to measure permeability directly at the outcrop and on cores. The operator presses a rubber nozzle against the surface where the reading is to be made and withdraws air from it with a single stroke of a syringe. As air is pulled from the sample, a microcontroller unit simultaneously monitors the syringe volume and the transient vacuum pulse created at the sample surface. Measurements are automatically stored and cataloged on a NER-supplied Android™ device that communicates wirelessly with the TinyPerm permeameter. The measurement data are captured in data files and stored in Darcy (D). They were later converted to the standard m^2^ permeability unit of measure (1 D = 9.869 x 10^-13^ m^2^) and calculated as log_10_ m^2^ to aid calculating minimum, maximum, and mean permeability values and their graphing.

|  | **Table S1. Summary of in situ permeability measurements along the Zuccale and Boccheggiano Fault** | | | | |
| --- | --- | --- | --- | --- | --- |
|  | Zuccale Fault | | | | |
| Number | Structural element | Permeability  (D) | Permeability  (m^2^) | Latitude  (UTM WGS84) | Longitude  (UTM WGS84) |
| 1 | Orthogonal to PSS (principal slip surface) | 7,7E-03 | 7,60E-15 | 42,75306252 N | 10,35716166 E |
| 2 | Orthogonal to PSS (principal slip surface) | 2,0E-03 | 1,97E-15 | 42,75305123 N | 10,35717833 E |
| 3 | Orthogonal to PSS (principal slip surface) | 1,7E-03 | 1,68E-15 | 42,75302126 N | 10,35719846 E |
| 4 | Orthogonal to PSS (principal slip surface) | 3,0E-03 | 2,96E-15 | 42,7530294 N | 10,35719257 E |
| 5 | Orthogonal to PSS (principal slip surface) | 9,5E-04 | 9,38E-16 | 42,7530294 N | 10,35719257 E |
| 6 | Orthogonal to PSS (principal slip surface) | 1,0E-01 | 9,87E-14 | 42,75303723 N | 10,35718368 E |
| 7 | Orthogonal to PSS (principal slip surface) | 1,8E-03 | 1,78E-15 | 42,75304821 N | 10,35715606 E |
| 8 | Orthogonal to PSS (principal slip surface) | 1,1E-01 | 1,09E-13 | 42,75304821 N | 10,35715606 E |
| 9 | Orthogonal to PSS (principal slip surface) | 3,9E-04 | 3,85E-16 | 42,75307447 N | 10,35714684 E |
| 10 | Orthogonal to PSS (principal slip surface) | 7,4E-04 | 7,30E-16 | 42,75307447 N | 10,35714684 E |
| 11 | Orthogonal to PSS (principal slip surface) | 2,7E-03 | 2,66E-15 | 42,75306242 N | 10,3571568 E |
| 12 | Orthogonal to PSS (principal slip surface) | 1,0E-01 | 9,87E-14 | 42,75305392 N | 10,35717719 E |
| 13 | Orthogonal to PSS (principal slip surface) | 6,7E-05 | 6,61E-17 | 42,75305097 N | 10,35717226 E |
| 14 | Orthogonal to PSS (principal slip surface) | 2,9E-03 | 2,86E-15 | 42,75305097 N | 10,35717226 E |
| 15 | Orthogonal to PSS (principal slip surface) | 9,9E-02 | 9,77E-14 | 42,75305215 N | 10,35717367 E |
| 16 | Orthogonal to PSS (principal slip surface) | 2,1E-03 | 2,07E-15 | 42,75305215 N | 10,35717367 E |
| 17 | Orthogonal to PSS (principal slip surface) | 4,7E-04 | 4,64E-16 | 42,75304828 N | 10,35717554 E |
| 18 | Orthogonal to PSS (principal slip surface) | 6,9E-05 | 6,81E-17 | 42,75305026 N | 10,35717315 E |
| 19 | Orthogonal to PSS (principal slip surface) | 1,5E-03 | 1,48E-15 | 42,75305112 N | 10,35717189 E |
| 20 | Orthogonal to PSS (principal slip surface) | 4,4E-02 | 4,34E-14 | 42,75305112 N | 10,35717189 E |
| 21 | Orthogonal to PSS (principal slip surface) | 9,8E-02 | 9,67E-14 | 42,75309375 N | 10,3571312 E |
| 22 | Orthogonal to PSS (principal slip surface) | 1,5E-03 | 1,48E-15 | 42,75309926 N | 10,35713792 E |
| 23 | Orthogonal to PSS (principal slip surface) | 9,2E-02 | 9,08E-14 | 42,75310909 N | 10,35713158 E |
| 24 | Orthogonal to PSS (principal slip surface) | 1,0E-01 | 9,87E-14 | 42,75310507 N | 10,3571419 E |
| 25 | Orthogonal to PSS (principal slip surface) | 6,3E-05 | 6,22E-17 | 42,75310507 N | 10,3571419 E |
| 26 | Orthogonal to PSS (principal slip surface) | 1,0E-01 | 9,87E-14 | 42,75309806 N | 10,35714276 E |
| 27 | Orthogonal to PSS (principal slip surface) | 3,5E-03 | 3,45E-15 | 42,75309611 N | 10,3571441 E |
| 28 | Orthogonal to PSS (principal slip surface) | 1,1E-01 | 1,09E-13 | 42,75309611 N | 10,3571441 E |
| 29 | Orthogonal to PSS (principal slip surface) | 9,3E-02 | 9,18E-14 | 42,75309898 N | 10,35714038 E |
| 30 | Orthogonal to PSS (principal slip surface) | 9,5E-04 | 9,38E-16 | 42,75309878 N | 10,35714096 E |
| 31 | Orthogonal to PSS (principal slip surface) | 1,0E-02 | 9,87E-15 | 42,75309878 N | 10,35714096 E |
| 32 | Orthogonal to PSS (principal slip surface) | 3,5E-02 | 3,45E-14 | 42,75309355 N | 10,35714942 E |
| 33 | Orthogonal to PSS (principal slip surface) | 1,1E-04 | 1,09E-16 | 42,75309046 N | 10,35715344 E |
| 34 | Orthogonal to PSS (principal slip surface) | 4,9E-03 | 4,84E-15 | 42,75308627 N | 10,35715284 E |
| 35 | Orthogonal to PSS (principal slip surface) | 1,1E-01 | 1,09E-13 | 42,75308627 N | 10,35715284 E |
| 36 | Orthogonal to PSS (principal slip surface) | 9,2E-04 | 9,08E-16 | 42,75308767 N | 10,35715198 E |
| 37 | Orthogonal to PSS (principal slip surface) | 2,9E-02 | 2,86E-14 | 42,75308335 N | 10,35715597 E |
| 38 | Orthogonal to PSS (principal slip surface) | 1,2E-02 | 1,18E-14 | 42,75308817 N | 10,35715726 E |
| 39 | Orthogonal to PSS (principal slip surface) | 1,5E-02 | 1,48E-14 | 42,75308607 N | 10,35715919 E |
| 40 | Orthogonal to PSS (principal slip surface) | 5,0E-04 | 4,93E-16 | 42,75308607 N | 10,35715919 E |
| 42 | Orthogonal to PSS (principal slip surface) | 9,7E-04 | 9,57E-16 | 42,75308607 N | 10,35715919 E |
| 43 | Orthogonal to PSS (principal slip surface) | 2,3E-03 | 2,27E-15 | 42,75308607 N | 10,35715919 E |
| 44 | Orthogonal to PSS (principal slip surface) | 9,0E-05 | 8,88E-17 | 42,75308607 N | 10,35715919 E |
| 45 | Orthogonal to PSS (principal slip surface) | 1,1E-01 | 1,09E-13 | 42,75308607 N | 10,35715919 E |
| 46 | Orthogonal to PSS (principal slip surface) | 1,5E-03 | 1,48E-15 | 42,75308607 N | 10,35715919 E |
| 47 | Orthogonal to PSS (principal slip surface) | 1,0E-01 | 9,87E-14 | 42,75308607 N | 10,35715919 E |
| 48 | Orthogonal to PSS (principal slip surface) | 2,5E-04 | 2,47E-16 | 42,75308607 N | 10,35715919 E |
| 49 | Orthogonal to PSS (principal slip surface) | 1,1E-01 | 1,09E-13 | 42,75308607 N | 10,35715919 E |
| 50 | Orthogonal to PSS (principal slip surface) | 3,0E-03 | 2,96E-15 | 42,75308607 N | 10,35715919 E |
| 51 | Orthogonal to PSS (principal slip surface) | 5,9E-04 | 5,82E-16 | 42,75308607 N | 10,35715919 E |
| 52 | Orthogonal to PSS (principal slip surface) | 7,4E-04 | 7,30E-16 | 42,75308607 N | 10,35715919 E |
| 53 | Orthogonal to PSS (principal slip surface) | 1,1E-03 | 1,09E-15 | 42,75308607 N | 10,35715919 E |
| 54 | Orthogonal to PSS (principal slip surface) | 6,3E-05 | 6,22E-17 | 42,75308607 N | 10,35715919 E |
| 55 | Orthogonal to PSS (principal slip surface) | 3,9E-03 | 3,85E-15 | 42,75308607 N | 10,35715919 E |
| 56 | Orthogonal to PSS (principal slip surface) | 3,9E-04 | 3,85E-16 | 42,75308607 N | 10,35715919 E |
| 57 | Orthogonal to PSS (principal slip surface) | 3,0E-03 | 2,96E-15 | 42,75308607 N | 10,35715919 E |
| 58 | Orthogonal to PSS (principal slip surface) | 1,1E-01 | 1,09E-13 | 42,75308607 N | 10,35715919 E |
| 59 | Orthogonal to PSS (principal slip surface) | 1,3E-01 | 1,28E-13 | 42,75308607 N | 10,35715919 E |
| 60 | Orthogonal to PSS (principal slip surface) | 1,5E-03 | 1,48E-15 | 42,75308607 N | 10,35715919 E |
| 61 | Orthogonal to PSS (principal slip surface) | 1,1E-03 | 1,09E-15 | 42,75308607 N | 10,35715919 E |
| 62 | Orthogonal to PSS (principal slip surface) | 4,8E-03 | 4,74E-15 | 42,75308607 N | 10,35715919 E |
|  |  | | | | |
| 1 | Parallel to foliation within BSF1 | 2,7E+00 | 2,66E-12 | 42,75304023 N | 10,35710752 E |
| 2 | Parallel to foliation within BSF1 | 1,1E+00 | 1,09E-12 | 42,75306463 N | 10,35723986 E |
| 3 | Parallel to foliation within BSF1 | 1,5E+00 | 1,48E-12 | 42,75306463 N | 10,35723986 E |
| 4 | Parallel to foliation within BSF1 | 1,1E+00 | 1,09E-12 | 42,75304682 N | 10,35709502 E |
| 5 | Parallel to foliation within BSF1 | 1,1E+00 | 1,09E-12 | 42,75304682 N | 10,35709502 E |
| 6 | Parallel to foliation within BSF1 | 1,1E+00 | 1,09E-12 | 42,75304682 N | 10,35709502 E |
| 7 | Parallel to foliation within BSF1 | 4,0E+00 | 3,95E-12 | 42,75298771 N | 10,3572003 E |
| 8 | Parallel to foliation within BSF1 | 3,8E+00 | 3,75E-12 | 42,75298771 N | 10,3572003 E |
| 9 | Parallel to foliation within BSF1 | 4,4E+00 | 4,34E-12 | 42,75298771 N | 10,3572003 E |
| 10 | Parallel to foliation within BSF1 | 1,5E+01 | 1,48E-11 | 42,75304003 N | 10,35713484 E |
| 11 | Parallel to foliation within BSF1 | 1,1E+01 | 1,09E-11 | 42,75304744 N | 10,35712763 E |
| 12 | Parallel to foliation within BSF1 | 8,6E+00 | 8,49E-12 | 42,75304744 N | 10,35712763 E |
| 13 | Parallel to foliation within BSF1 | 2,0E+01 | 1,97E-11 | 42,75305177 N | 10,35714335 E |
|  |  | | | | |
| 1 | Parallel to elongated sigmoidal lithons within foliations in the Cretaceous flysch | 7,9E-01 | 7,80E-13 | 42,7530524 N | 10,35714405 E |
| 2 | Parallel to elongated sigmoidal lithons within foliations in the Cretaceous flysch | 1,6E+00 | 1,58E-12 | 42,7530524 N | 10,35714405 E |
| 3 | Parallel to elongated sigmoidal lithons within foliations in the Cretaceous flysch | 1,6E+00 | 1,58E-12 | 42,7530524 N | 10,35714405 E |
|  |  | | | | |
| 1 | Parallel to foliation within the Cretaceous flysch | 4,9E+00 | 4,84E-12 | 42,7530524 N | 10,35714405 E |
| 2 | Parallel to foliation within the Cretaceous flysch | 4,9E+00 | 4,84E-12 | 42,7530524 N | 10,35714405 E |
| 3 | Parallel to foliation within the Cretaceous flysch | 2,8E+00 | 2,76E-12 | 42,7530524 N | 10,35714405 E |
| 4 | Parallel to foliation within the Cretaceous flysch | 3,2E+00 | 3,16E-12 | 42,7530524 N | 10,35714405 E |
| 5 | Parallel to foliation within the Cretaceous flysch | 3,3E+00 | 3,26E-12 | 42,7530524 N | 10,35714405 E |
| 6 | Parallel to foliation within the Cretaceous flysch | 3,6E+00 | 3,55E-12 | 42,7530524 N | 10,35714405 E |
| 7 | Parallel to foliation within the Cretaceous flysch | 1,2E+01 | 1,18E-11 | 42,7530524 N | 10,35714405 E |
| 8 | Parallel to foliation within the Cretaceous flysch | 8,4E+00 | 8,29E-12 | 42,7530524 N | 10,35714405 E |
| 9 | Parallel to foliation within the Cretaceous flysch | 9,9E+00 | 9,77E-12 | 42,7530524 N | 10,35714405 E |
| 10 | Parallel to foliation within the Cretaceous flysch | 1,2E+01 | 1,18E-11 | 42,7530524 N | 10,35714405 E |
| 11 | Parallel to foliation within the Cretaceous flysch | 9,6E+00 | 9,47E-12 | 42,7530524 N | 10,35714405 E |
| 12 | Parallel to foliation within the Cretaceous flysch | 9,2E+00 | 9,08E-12 | 42,7530524 N | 10,35714405 E |
|  |  | | | | |
| 1 | Orthogonal to foliation within the Cretaceous flysch | 2,5E-02 | 2,47E-14 | 42,75287965 N | 10,35705349 E |
| 2 | Orthogonal to foliation within the Cretaceous flysch | 6,5E-02 | 6,41E-14 | 42,75291608 N | 10,3570337 E |
| 3 | Orthogonal to foliation within the Cretaceous flysch | 3,4E-02 | 3,36E-14 | 42,75291608 N | 10,3570337 E |
| 4 | Orthogonal to foliation within the Cretaceous flysch | 1,6E-02 | 1,58E-14 | 42,75291608 N | 10,3570337 E |
| 5 | Orthogonal to foliation within the Cretaceous flysch | 5,9E-02 | 5,82E-14 | 42,75294769 N | 10,35705382 E |
| 6 | Orthogonal to foliation within the Cretaceous flysch | 3,1E-02 | 3,06E-14 | 42,75294769 N | 10,35705382 E |
| 7 | Orthogonal to foliation within the Cretaceous flysch | 4,9E-01 | 4,84E-13 | 42,75294769 N | 10,35705382 E |
| 8 | Orthogonal to foliation within the Cretaceous flysch | 5,3E-01 | 5,23E-13 | 42,75294769 N | 10,35705382 E |
| 9 | Orthogonal to foliation within the Cretaceous flysch | 2,2E-01 | 2,17E-13 | 42,75295974 N | 10,35707274 E |
| 10 | Orthogonal to foliation within the Cretaceous flysch | 5,5E-02 | 5,43E-14 | 42,75296423 N | 10,3571031 E |
| 11 | Orthogonal to foliation within the Cretaceous flysch | 5,2E-02 | 5,13E-14 | 42,75296878 N | 10,3570988 E |
| 12 | Orthogonal to foliation within the Cretaceous flysch | 4,8E-02 | 4,74E-14 | 42,75296878 N | 10,3570988 E |
| 13 | Orthogonal to foliation within the Cretaceous flysch | 1,7E-01 | 1,68E-13 | 42,75297476 N | 10,35710511 E |
| 14 | Orthogonal to foliation within the Cretaceous flysch | 7,4E-02 | 7,30E-14 | 42,75297476 N | 10,35710511 E |
| 15 | Orthogonal to foliation within the Cretaceous flysch | 7,2E-02 | 7,11E-14 | 42,75297727 N | 10,35711309 E |
| 16 | Orthogonal to foliation within the Cretaceous flysch | 9,0E-01 | 8,88E-13 | 42,75297727 N | 10,35711309 E |
| 17 | Orthogonal to foliation within the Cretaceous flysch | 8,1E-01 | 7,99E-13 | 42,75297908 N | 10,35711593 E |
| 18 | Orthogonal to foliation within the Cretaceous flysch | 8,6E-01 | 8,49E-13 | 42,75297908 N | 10,35711593 E |
| 19 | Orthogonal to foliation within the Cretaceous flysch | 4,1E-03 | 4,05E-15 | 42,75298074 N | 10,35711443 E |
| 20 | Orthogonal to foliation within the Cretaceous flysch | 8,2E-03 | 8,09E-15 | 42,75298074 N | 10,35711443 E |
| 21 | Orthogonal to foliation within the Cretaceous flysch | 6,1E-03 | 6,02E-15 | 42,75298262 N | 10,35711426 E |
| 22 | Orthogonal to foliation within the Cretaceous flysch | 7,2E-03 | 7,11E-15 | 42,75298262 N | 10,35711426 E |
| 23 | Orthogonal to foliation within the Cretaceous flysch | 8,3E-03 | 8,19E-15 | 42,75299774 N | 10,35711348 E |
| 24 | Orthogonal to foliation within the Cretaceous flysch | 2,2E-03 | 2,17E-15 | 42,75299774 N | 10,35711348 E |
| 25 | Orthogonal to foliation within the Cretaceous flysch | 1,7E-02 | 1,68E-14 | 42,75298144 N | 10,35711789 E |
| 26 | Orthogonal to foliation within the Cretaceous flysch | 3,0E-02 | 2,96E-14 | 42,75298144 N | 10,35711789 E |
| 27 | Orthogonal to foliation within the Cretaceous flysch | 1,2E-02 | 1,18E-14 | 42,75299109 N | 10,35711494 E |
| 28 | Orthogonal to foliation within the Cretaceous flysch | 1,1E+00 | 1,09E-12 | 42,75299109 N | 10,35711494 E |
| 29 | Orthogonal to foliation within the Cretaceous flysch | 9,8E-01 | 9,67E-13 | 42,75299006 N | 10,35710141 E |
| 30 | Orthogonal to foliation within the Cretaceous flysch | 8,0E-01 | 7,90E-13 | 42,75299006 N | 10,35710141 E |
|  |  | | | | |
| 1 | Parallel to foliation within the poorly foliated quartzites of the Verrucano Fm. | 5,3E-01 | 5,23E-13 | 42,75252462 N | 10,35717398 E |
| 2 | Parallel to foliation within the poorly foliated quartzites of the Verrucano Fm. | 1,1E+00 | 1,09E-12 | 42,75252462 N | 10,35717398 E |
| 3 | Parallel to foliation within the poorly foliated quartzites of the Verrucano Fm. | 2,6E-02 | 2,57E-14 | 42,75252462 N | 10,35717398 E |
| 4 | Parallel to foliation within the poorly foliated quartzites of the Verrucano Fm. | 8,1E-03 | 7,99E-15 | 42,75252462 N | 10,35717398 E |
| 5 | Parallel to foliation within the poorly foliated quartzites of the Verrucano Fm. | 5,6E-01 | 5,53E-13 | 42,75250717 N | 10,35718394 E |
| 6 | Parallel to foliation within the poorly foliated quartzites of the Verrucano Fm. | 5,0E-01 | 4,93E-13 | 42,75250717 N | 10,35718394 E |
|  |  | | | | |
| 1 | Parallel to foliation within the highly foliated quartzites of the Verrucano Fm. | 1,9E-03 | 1,88E-15 | 42,75250717 N | 10,35718394 E |
| 2 | Parallel to foliation within the highly foliated quartzites of the Verrucano Fm. | 1,5E-03 | 1,48E-15 | 42,75250717 N | 10,35718394 E |
| 3 | Parallel to foliation within the highly foliated quartzites of the Verrucano Fm. | 4,2E-03 | 4,14E-15 | 42,75250717 N | 10,35718394 E |
| 4 | Parallel to foliation within the highly foliated quartzites of the Verrucano Fm. | 1,2E-01 | 1,18E-13 | 42,75250717 N | 10,35718394 E |
| 5 | Parallel to foliation within the highly foliated quartzites of the Verrucano Fm. | 2,4E-03 | 2,37E-15 | 42,75250717 N | 10,35718394 E |
| 6 | Parallel to foliation within the highly foliated quartzites of the Verrucano Fm. | 3,7E-01 | 3,65E-13 | 42,75250717 N | 10,35718394 E |
| 7 | Parallel to foliation within the highly foliated quartzites of the Verrucano Fm. | 5,4E-04 | 5,33E-16 | 42,75250717 N | 10,35718394 E |
| 8 | Parallel to foliation within the highly foliated quartzites of the Verrucano Fm. | 7,2E-01 | 7,11E-13 | 42,75250717 N | 10,35718394 E |
| 9 | Parallel to foliation within the highly foliated quartzites of the Verrucano Fm. | 5,3E+00 | 5,23E-12 | 42,75250717 N | 10,35718394 E |
| 10 | Parallel to foliation within the highly foliated quartzites of the Verrucano Fm. | 1,1E+00 | 1,09E-12 | 42,75250717 N | 10,35718394 E |
|  |  | | | | |
| 1 | Parallel to foliation within the calc-mylonitic marble | 6,2E+00 | 6,12E-12 | 42,75250037 N | 10,35713058 E |
| 2 | Parallel to foliation within the calc-mylonitic marble | 7,7E+00 | 7,60E-12 | 42,75250037 N | 10,35713058 E |
| 3 | Parallel to foliation within the calc-mylonitic marble | 8,9E+00 | 8,78E-12 | 42,75250037 N | 10,35713058 E |
| 4 | Parallel to foliation within the calc-mylonitic marble | 2,4E+00 | 2,37E-12 | 42,75250142 N | 10,35712855 E |
| 5 | Parallel to foliation within the calc-mylonitic marble | 1,7E+00 | 1,68E-12 | 42,75250142 N | 10,35712855 E |
| 6 | Parallel to foliation within the calc-mylonitic marble | 9,4E+00 | 9,28E-12 | 42,75250142 N | 10,35712855 E |
| 7 | Parallel to foliation within the calc-mylonitic marble | 3,4E+00 | 3,36E-12 | 42,75250142 N | 10,35712855 E |
| 8 | Parallel to foliation within the calc-mylonitic marble | 5,8E+00 | 5,72E-12 | 42,75248169 N | 10,35709457 E |
| 9 | Parallel to foliation within the calc-mylonitic marble | 3,4E+00 | 3,36E-12 | 42,75248169 N | 10,35709457 E |
| 10 | Parallel to foliation within the calc-mylonitic marble | 6,0E+00 | 5,92E-12 | 42,7524859 N | 10,35712774 E |
| 11 | Parallel to foliation within the calc-mylonitic marble | 2,7E+00 | 2,66E-12 | 42,7524859 N | 10,35712774 E |
| 12 | Parallel to foliation within the calc-mylonitic marble | 1,6E+00 | 1,58E-12 | 42,7524859 N | 10,35712774 E |
| 13 | Parallel to foliation within the calc-mylonitic marble | 5,2E-01 | 5,13E-13 | 42,7524859 N | 10,35712774 E |
| 14 | Parallel to foliation within the calc-mylonitic marble | 1,7E+00 | 1,68E-12 | 42,7524859 N | 10,35712774 E |
| 15 | Parallel to foliation within the calc-mylonitic marble | 4,2E-01 | 4,14E-13 | 42,7524859 N | 10,35712774 E |
| 16 | Parallel to foliation within the calc-mylonitic marble | 6,3E+00 | 6,22E-12 | 42,75245899 N | 10,35707954 E |
| 17 | Parallel to foliation within the calc-mylonitic marble | 1,0E+01 | 9,87E-12 | 42,75245899 N | 10,35707954 E |
| 18 | Parallel to foliation within the calc-mylonitic marble | 7,0E+00 | 6,91E-12 | 42,75245899 N | 10,35707954 E |
| 19 | Parallel to foliation within the calc-mylonitic marble | 4,3E+00 | 4,24E-12 | 42,75244984 N | 10,3570821 E |
| 20 | Parallel to foliation within the calc-mylonitic marble | 4,6E+00 | 4,54E-12 | 42,75244984 N | 10,3570821 E |
| 21 | Parallel to foliation within the calc-mylonitic marble | 3,7E+00 | 3,65E-12 | 42,75244984 N | 10,3570821 E |
| 22 | Parallel to foliation within the calc-mylonitic marble | 4,6E+00 | 4,54E-12 | 42,75247655 N | 10,35704596 E |
| 23 | Parallel to foliation within the calc-mylonitic marble | 4,0E+00 | 3,95E-12 | 42,75247655 N | 10,35704596 E |
| 24 | Parallel to foliation within the calc-mylonitic marble | 4,9E+00 | 4,84E-12 | 42,75247655 N | 10,35704596 E |
|  |  | | | | |
| 1 | Orthogonal to foliation within the calc-mylonitic marble | 2,5E-04 | 2,47E-16 | 42,75248384 N | 10,35706837 E |
| 2 | Orthogonal to foliation within the calc-mylonitic marble | 1,9E-04 | 1,88E-16 | 42,75248384 N | 10,35706837 E |
| 3 | Orthogonal to foliation within the calc-mylonitic marble | 3,5E-04 | 3,45E-16 | 42,75248258 N | 10,35706756 E |
| 4 | Orthogonal to foliation within the calc-mylonitic marble | 5,1E-04 | 5,03E-16 | 42,75248258 N | 10,35706756 E |
| 5 | Orthogonal to foliation within the calc-mylonitic marble | 6,3E-04 | 6,22E-16 | 42,75248258 N | 10,35706756 E |
| 6 | Orthogonal to foliation within the calc-mylonitic marble | 1,9E-04 | 1,88E-16 | 42,75247579 N | 10,35707387 E |
| 7 | Orthogonal to foliation within the calc-mylonitic marble | 4,4E-03 | 4,34E-15 | 42,75248379 N | 10,35707407 E |
| 8 | Orthogonal to foliation within the calc-mylonitic marble | 3,6E-03 | 3,55E-15 | 42,75248846 N | 10,35708269 E |
| 9 | Orthogonal to foliation within the calc-mylonitic marble | 6,1E-03 | 6,02E-15 | 42,75248858 N | 10,35707594 E |
| 10 | Orthogonal to foliation within the calc-mylonitic marble | 1,6E-02 | 1,58E-14 | 42,75248684 N | 10,3570788 E |
| 11 | Orthogonal to foliation within the calc-mylonitic marble | 3,0E-02 | 2,96E-14 | 42,75248684 N | 10,3570788 E |
| 12 | Orthogonal to foliation within the calc-mylonitic marble | 2,0E-02 | 1,97E-14 | 42,75248684 N | 10,3570788 E |
| 13 | Orthogonal to foliation within the calc-mylonitic marble | 8,4E-03 | 8,29E-15 | 42,75248472 N | 10,35707965 E |
| 14 | Orthogonal to foliation within the calc-mylonitic marble | 1,0E-02 | 9,87E-15 | 42,75248472 N | 10,35707965 E |
| 15 | Orthogonal to foliation within the calc-mylonitic marble | 4,5E-03 | 4,44E-15 | 42,75248679 N | 10,35707546 E |
| 16 | Orthogonal to foliation within the calc-mylonitic marble | 5,9E-01 | 5,82E-13 | 42,75248679 N | 10,35707546 E |
| 17 | Orthogonal to foliation within the calc-mylonitic marble | 3,5E-03 | 3,45E-15 | 42,75248599 N | 10,35707611 E |
| 18 | Orthogonal to foliation within the calc-mylonitic marble | 5,0E-03 | 4,93E-15 | 42,75248613 N | 10,35708202 E |
| 19 | Orthogonal to foliation within the calc-mylonitic marble | 2,1E-02 | 2,07E-14 | 42,75248613 N | 10,35708202 E |
| 20 | Orthogonal to foliation within the calc-mylonitic marble | 1,8E-02 | 1,78E-14 | 42,75248649 N | 10,35708236 E |
| 21 | Orthogonal to foliation within the calc-mylonitic marble | 2,2E-02 | 2,17E-14 | 42,75249281 N | 10,35708424 E |
|  |  | | | | |
| 1 | Parallel to foliation within BSF3a | 3,6E+00 | 3,55E-12 | 42,7522405 N | 10,35732191 E |
| 2 | Parallel to foliation within BSF3a | 5,3E+00 | 5,23E-12 | 42,75223104 N | 10,35732191 E |
| 3 | Parallel to foliation within BSF3a | 3,9E+00 | 3,85E-12 | 42,75223104 N | 10,35732191 E |
| 4 | Parallel to foliation within BSF3a | 3,7E+00 | 3,65E-12 | 42,75223104 N | 10,35732191 E |
| 5 | Parallel to foliation within BSF3a | 1,6E+00 | 1,58E-12 | 42,75223104 N | 10,35732191 E |
| 6 | Parallel to foliation within BSF3a | 9,7E-01 | 9,57E-13 | 42,75223104 N | 10,35732191 E |
| 7 | Parallel to foliation within BSF3a | 3,8E+00 | 3,75E-12 | 42,75223104 N | 10,35732191 E |
| 8 | Parallel to foliation within BSF3a | 8,8E-01 | 8,68E-13 | 42,75223104 N | 10,35732191 E |
| 9 | Parallel to foliation within BSF3a | 7,6E-01 | 7,50E-13 | 42,75223104 N | 10,35732191 E |
|  |  | | | | |
| 1 | Parallel to foliation within BSF3b | 6,8E+00 | 6,71E-12 | 42,75223104 N | 10,35732191 E |
| 2 | Parallel to foliation within BSF3b | 6,6E+00 | 6,51E-12 | 42,75223104 N | 10,35732191 E |
| 3 | Parallel to foliation within BSF3b | 3,7E+00 | 3,65E-12 | 42,75223104 N | 10,35732191 E |
| 4 | Parallel to foliation within BSF3b | 2,1E-01 | 2,07E-13 | 42,75223104 N | 10,35732191 E |
| 5 | Parallel to foliation within BSF3b | 3,4E-01 | 3,36E-13 | 42,75223104 N | 10,35732191 E |
| 6 | Parallel to foliation within BSF3b | 1,6E-01 | 1,58E-13 | 42,75223104 N | 10,35732191 E |
| 7 | Parallel to foliation within BSF3b | 8,7E-02 | 8,59E-14 | 42,75223104 N | 10,35732191 E |
| 8 | Parallel to foliation within BSF3b | 5,3E-01 | 5,23E-13 | 42,75223104 N | 10,35732191 E |
| 9 | Parallel to foliation within BSF3b | 1,3E-01 | 1,28E-13 | 42,75223104 N | 10,35732191 E |
|  |  | | | | |
| 1 | Parallel to foliation within BSF3c | 2,4E+00 | 2,37E-12 | 42,75223104 N | 10,35732191 E |
| 2 | Parallel to foliation within BSF3c | 5,4E-01 | 5,33E-13 | 42,75223104 N | 10,35732191 E |
| 3 | Parallel to foliation within BSF3c | 7,0E-01 | 6,91E-13 | 42,75223104 N | 10,35732191 E |
|  | Boccheggiano Fault | | | | |
| 1 | Orthogonal to So of the Argille a Palombini Fm. | 1,6E-01 | 1,58E-13 | 43.09884992 N | 11.03333726 E |
| 2 | Orthogonal to So of the Argille a Palombini Fm. | 9,4E-02 | 9,28E-14 | 43.09879263 N | 11.03346697 E |
| 3 | Orthogonal to So of the Argille a Palombini Fm. | 6,9E-02 | 6,81E-14 | 43.09879263 N | 11.03346697 E |
| 4 | Orthogonal to So of the Argille a Palombini Fm. | 2,6E-01 | 2,57E-13 | 43.09862665 N | 11.03344934 E |
| 5 | Orthogonal to So of the Argille a Palombini Fm. | 1,7E+00 | 1,68E-12 | 43.09884992 N | 11.03333726 E |
| 6 | Orthogonal to So of the Argille a Palombini Fm. | 1,4E+00 | 1,38E-12 | 43.09884992 N | 11.03333726 E |
| 7 | Orthogonal to So of the Argille a Palombini Fm. | 1,4E+00 | 1,38E-12 | 43.09879263 N | 11.03346697 E |
| 8 | Orthogonal to So of the Argille a Palombini Fm. | 6,2E-02 | 6,12E-14 | 43.09862201 N | 11.03345879 E |
| 9 | Orthogonal to So of the Argille a Palombini Fm. | 7,7E-02 | 7,60E-14 | 43.09861825 N | 11.0334521 E |
| 10 | Orthogonal to So of the Argille a Palombini Fm. | 3,6E-01 | 3,55E-13 | 43.09861825 N | 11.0334521 E |
|  |  | | | | |
| 1 | Parallel to So of the Argille a Palombini Fm. | 6,0E-03 | 5,92E-15 | 43.09868262 N | 11.03353188 E |
| 2 | Parallel to So of the Argille a Palombini Fm. | 1,9E-03 | 1,88E-15 | 43.09863476 N | 11.03349045 E |
| 3 | Parallel to So of the Argille a Palombini Fm. | 3,0E-03 | 2,96E-15 | 43.09862969 N | 11.03347366 E |
| 4 | Parallel to So of the Argille a Palombini Fm. | 8,8E-04 | 8,68E-16 | 43.09861463 N | 11.03345226 E |
| 5 | Parallel to So of the Argille a Palombini Fm. | 3,1E-03 | 3,06E-15 | 43.09861463 N | 11.03345226 E |
| 6 | Parallel to So of the Argille a Palombini Fm. | 4,3E-03 | 4,24E-15 | 43.0986197 N | 11.03344778 E |
| 7 | Parallel to So of the Argille a Palombini Fm. | 4,2E-04 | 4,14E-16 | 43.09862665 N | 11.03344934 E |
| 8 | Parallel to So of the Argille a Palombini Fm. | 1,0E-03 | 9,87E-16 | 43.09862665 N | 11.03344934 E |
| 9 | Parallel to So of the Argille a Palombini Fm. | 4,9E-04 | 4,84E-16 | 43.09884992 N | 11.03333726 E |
|  |  | | | | |
| 1 | BSF1 | 6,0E-03 | 5,92E-15 | 43.09868262 N | 11.03353188 E |
| 2 | BSF1 | 3,5E-03 | 3,45E-15 | 43.09863476 N | 11.03349045 E |
| 3 | BSF1 | 7,1E-03 | 7,01E-15 | 43.09862969 N | 11.03347366 E |
| 4 | BSF1 | 1,8E-02 | 1,78E-14 | 43.09862201 N | 11.03345879 E |
| 5 | BSF1 | 1,8E-02 | 1,78E-14 | 43.09861825 N | 11.0334521 E |
| 6 | BSF1 | 4,2E-02 | 4,14E-14 | 43.09861825 N | 11.0334521 E |
| 7 | BSF1 | 1,0E+00 | 9,87E-13 | 43.09861463 N | 11.03345226 E |
| 8 | BSF1 | 6,9E-04 | 6,81E-16 | 43.09861463 N | 11.03345226 E |
| 9 | BSF1 | 2,3E-03 | 2,27E-15 | 43.0986197 N | 11.03344778 E |
| 10 | BSF1 | 1,5E-03 | 1,48E-15 | 43.09862665 N | 11.03344934 E |
| 11 | BSF1 | 3,0E-02 | 2,96E-14 | 43.09862665 N | 11.03344934 E |
| 12 | BSF1 | 2,7E-04 | 2,66E-16 | 43.09884992 N | 11.03333726 E |
| 13 | BSF1 | 2,5E-03 | 2,47E-15 | 43.09884992 N | 11.03333726 E |
| 14 | BSF1 | 1,6E-01 | 1,58E-13 | 43.09879263 N | 11.03346697 E |
| 15 | BSF1 | 9,9E-04 | 9,77E-16 | 43.09879263 N | 11.03346697 E |
|  |  | | | | |
| 1 | Parallel to foliation within BSF2 | 3,3E+00 | 3,26E-12 | 43.09862275 N | 11.03332939 E |
| 2 | Parallel to foliation within BSF2 | 3,6E+00 | 3,55E-12 | 43.09862275 N | 11.03332939 E |
| 3 | Parallel to foliation within BSF2 | 2,5E+00 | 2,47E-12 | 43.09862275 N | 11.03332939 E |
| 4 | Parallel to foliation within BSF2 | 3,5E+00 | 3,45E-12 | 43.09862275 N | 11.03332939 E |
| 5 | Parallel to foliation within BSF2 | 5,0E+00 | 4,93E-12 | 43.09862275 N | 11.03332939 E |
| 6 | Parallel to foliation within BSF2 | 6,0E+00 | 5,92E-12 | 43.09862275 N | 11.03332939 E |
| 7 | Parallel to foliation within BSF2 | 2,2E+00 | 2,17E-12 | 43.09862275 N | 11.03332939 E |
| 8 | Parallel to foliation within BSF2 | 4,9E+00 | 4,84E-12 | 43.09862275 N | 11.03332939 E |
| 9 | Parallel to foliation within BSF2 | 2,4E+00 | 2,37E-12 | 43.09862275 N | 11.03332939 E |
| 10 | Parallel to foliation within BSF2 | 5,2E+00 | 5,13E-12 | 43.09862275 N | 11.03332939 E |
| 11 | Parallel to foliation within BSF2 | 2,8E+00 | 2,76E-12 | 43.09862275 N | 11.03332939 E |
| 12 | Parallel to foliation within BSF2 | 4,9E+00 | 4,84E-12 | 43.09862275 N | 11.03332939 E |
| 13 | Parallel to foliation within BSF2 | 2,8E+00 | 2,76E-12 | 43.09862275 N | 11.03332939 E |
| 14 | Parallel to foliation within BSF2 | 7,6E-01 | 7,50E-13 | 43.09862275 N | 11.03332939 E |
| 15 | Parallel to foliation within BSF2 | 2,8E-01 | 2,76E-13 | 43.09862275 N | 11.03332939 E |
| 16 | Parallel to foliation within BSF2 | 1,7E+01 | 1,68E-11 | 43.09862275 N | 11.03332939 E |
| 17 | Parallel to foliation within BSF2 | 6,4E+00 | 6,32E-12 | 43.09862275 N | 11.03332939 E |
| 18 | Parallel to foliation within BSF2 | 1,6E+01 | 1,58E-11 | 43.09862275 N | 11.03332939 E |
|  |  | | | | |
| 1 | BSF3 measurements collected progressively toward the PSS (from 1 to 26) | 7,70E-01 | 7,60E-13 | 43.09866596 N | 11.03334908 E |
| 2 | BSF3 measurements collected progressively toward the PSS (from 1 to 26) | 2,84E-01 | 2,80E-13 | 43.09866596 N | 11.03334908 E |
| 3 | BSF3 measurements collected progressively toward the PSS (from 1 to 26) | 8,05E-01 | 7,94E-13 | 43.09866596 N | 11.03334908 E |
| 4 | BSF3 measurements collected progressively toward the PSS (from 1 to 26) | 4,49E-02 | 4,43E-14 | 43.09866596 N | 11.03334908 E |
| 5 | BSF3 measurements collected progressively toward the PSS (from 1 to 26) | 7,45E-02 | 7,35E-14 | 43.09866596 N | 11.03334908 E |
| 6 | BSF3 measurements collected progressively toward the PSS (from 1 to 26) | 2,41E-01 | 2,38E-13 | 43.09866596 N | 11.03334908 E |
| 7 | BSF3 measurements collected progressively toward the PSS (from 1 to 26) | 1,48E-01 | 1,46E-13 | 43.09866596 N | 11.03334908 E |
| 8 | BSF3 measurements collected progressively toward the PSS (from 1 to 26) | 6,68E-04 | 6,59E-16 | 43.09866596 N | 11.03334908 E |
| 9 | BSF3 measurements collected progressively toward the PSS (from 1 to 26) | 9,31E-04 | 9,19E-16 | 43.09866596 N | 11.03334908 E |
| 10 | BSF3 measurements collected progressively toward the PSS (from 1 to 26) | 4,50E-02 | 4,44E-14 | 43.09866596 N | 11.03334908 E |
| 11 | BSF3 measurements collected progressively toward the PSS (from 1 to 26) | 1,06E-03 | 1,05E-15 | 43.09866596 N | 11.03334908 E |
| 12 | BSF3 measurements collected progressively toward the PSS (from 1 to 26) | 4,36E-03 | 4,30E-15 | 43.09866596 N | 11.03334908 E |
| 13 | BSF3 measurements collected progressively toward the PSS (from 1 to 26) | 5,25E-03 | 5,18E-15 | 43.09866596 N | 11.03334908 E |
| 14 | BSF3 measurements collected progressively toward the PSS (from 1 to 26) | 1,30E-02 | 1,28E-14 | 43.09866596 N | 11.03334908 E |
| 15 | BSF3 measurements collected progressively toward the PSS (from 1 to 26) | 4,58E-03 | 4,52E-15 | 43.09866596 N | 11.03334908 E |
| 16 | BSF3 measurements collected progressively toward the PSS (from 1 to 26) | 3,80E+00 | 3,75E-12 | 43.09866596 N | 11.03334908 E |
| 17 | BSF3 measurements collected progressively toward the PSS (from 1 to 26) | 2,02E+00 | 1,99E-12 | 43.09866596 N | 11.03334908 E |
| 18 | BSF3 measurements collected progressively toward the PSS (from 1 to 26) | 3,30E+00 | 3,26E-12 | 43.09866596 N | 11.03334908 E |
| 19 | BSF3 measurements collected progressively toward the PSS (from 1 to 26) | 1,86E+00 | 1,84E-12 | 43.09866596 N | 11.03334908 E |
| 20 | BSF3 measurements collected progressively toward the PSS (from 1 to 26) | 2,72E+00 | 2,68E-12 | 43.09866596 N | 11.03334908 E |
| 21 | BSF3 measurements collected progressively toward the PSS (from 1 to 26) | 5,34E-01 | 5,27E-13 | 43.09866596 N | 11.03334908 E |
| 22 | BSF3 measurements collected progressively toward the PSS (from 1 to 26) | 1,30E+00 | 1,28E-12 | 43.09866596 N | 11.03334908 E |
| 23 | BSF3 measurements collected progressively toward the PSS (from 1 to 26) | 3,20E-01 | 3,16E-13 | 43.09866596 N | 11.03334908 E |
| 24 | BSF3 measurements collected progressively toward the PSS (from 1 to 26) | 2,31E-01 | 2,28E-13 | 43.09866596 N | 11.03334908 E |
| 25 | BSF3 measurements collected progressively toward the PSS (from 1 to 26) | 1,38E-01 | 1,36E-13 | 43.09866596 N | 11.03334908 E |
| 26 | BSF3 measurements collected progressively toward the PSS (from 1 to 26) | 1,27E-02 | 1,25E-14 | 43.09866596 N | 11.03334908 E |
|  |  | | | | |
| 1 | Parallel to shear fractures within BSF4 | 4,1E-02 | 4,05E-14 | 42,75223104 N | 10,35727897 E |
| 2 | Parallel to shear fractures within BSF4 | 5,9E-01 | 5,82E-13 | 42,75223104 N | 10,35727897 E |
| 3 | Parallel to shear fractures within BSF4 | 6,0E-04 | 5,92E-16 | 42,75223104 N | 10,35727897 E |
| 4 | Parallel to shear fractures within BSF4 | 1,1E+00 | 1,09E-12 | 42,75223104 N | 10,35727897 E |
| 5 | Parallel to shear fractures within BSF4 | 5,7E-01 | 5,63E-13 | 42,75223104 N | 10,35727897 E |
| 6 | Parallel to shear fractures within BSF4 | 4,8E-01 | 4,74E-13 | 42,75223104 N | 10,35727897 E |
| 7 | Parallel to shear fractures within BSF4 | 4,3E+00 | 4,24E-12 | 42,75223104 N | 10,35727897 E |
| 8 | Parallel to shear fractures within BSF4 | 1,4E-01 | 1,38E-13 | 42,75223104 N | 10,35727897 E |
| 9 | Parallel to shear fractures within BSF4 | 5,5E+00 | 5,43E-12 | 42,75223104 N | 10,35727897 E |
| 10 | Parallel to shear fractures within BSF4 | 1,4E-03 | 1,38E-15 | 42,75223104 N | 10,35727897 E |
| 11 | Parallel to shear fractures within BSF4 | 2,5E-02 | 2,47E-14 | 42,75223104 N | 10,35727897 E |
| 12 | Parallel to shear fractures within BSF4 | 1,5E-03 | 1,48E-15 | 42,75223104 N | 10,35727897 E |
| 13 | Parallel to shear fractures within BSF4 | 2,3E-03 | 2,27E-15 | 42,75223104 N | 10,35727897 E |
| 14 | Parallel to shear fractures within BSF4 | 1,2E-01 | 1,18E-13 | 42,75223104 N | 10,35727897 E |
| 15 | Parallel to shear fractures within BSF4 | 2,7E-02 | 2,66E-14 | 42,75223104 N | 10,35727897 E |
| 16 | Parallel to shear fractures within BSF4 | 1,4E-01 | 1,38E-13 | 42,75223104 N | 10,35727897 E |
| 17 | Parallel to shear fractures within BSF4 | 2,8E-03 | 2,76E-15 | 42,75223104 N | 10,35727897 E |
| 18 | Parallel to shear fractures within BSF4 | 4,3E-02 | 4,24E-14 | 42,75223104 N | 10,35727897 E |
| 19 | Parallel to shear fractures within BSF4 | 1,2E-03 | 1,18E-15 | 42,75223104 N | 10,35727897 E |
|  |  | | | | |
| 1 | Orthogonal to foliation within the Paleozoic basement | 1,6E-03 | 1,58E-15 | 43.09859375 N | 11.03340941 E |
| 2 | Orthogonal to foliation within the Paleozoic basement | 1,8E-03 | 1,78E-15 | 43.09859375 N | 11.03340941 E |
| 3 | Orthogonal to foliation within the Paleozoic basement | 3,0E-03 | 2,96E-15 | 43.09859375 N | 11.03340941 E |
| 4 | Orthogonal to foliation within the Paleozoic basement | 8,1E-05 | 7,99E-17 | 43.09859375 N | 11.03340941 E |
| 5 | Orthogonal to foliation within the Paleozoic basement | 1,1E-03 | 1,09E-15 | 43.09859375 N | 11.03340941 E |
| 6 | Orthogonal to foliation within the Paleozoic basement | 5,8E-04 | 5,72E-16 | 43.09859375 N | 11.03340941 E |
| 7 | Orthogonal to foliation within the Paleozoic basement | 3,5E-04 | 3,45E-16 | 43.09859375 N | 11.03340941 E |
| 8 | Orthogonal to foliation within the Paleozoic basement | 2,5E-04 | 2,47E-16 | 43.09859375 N | 11.03340941 E |
| 9 | Orthogonal to foliation within the Paleozoic basement | 8,4E-02 | 8,29E-14 | 43.09859375 N | 11.03340941 E |
| 10 | Orthogonal to foliation within the Paleozoic basement | 6,0E-04 | 5,92E-16 | 43.09859375 N | 11.03340941 E |
| 11 | Orthogonal to foliation within the Paleozoic basement | 8,7E-02 | 8,59E-14 | 43.09859375 N | 11.03340941 E |
| 12 | Orthogonal to foliation within the Paleozoic basement | 2,8E-04 | 2,76E-16 | 43.09859375 N | 11.03340941 E |
| 13 | Orthogonal to foliation within the Paleozoic basement | 8,5E-02 | 8,39E-14 | 43.09859375 N | 11.03340941 E |
| 14 | Orthogonal to foliation within the Paleozoic basement | 7,7E-04 | 7,60E-16 | 43.09859375 N | 11.03340941 E |
| 15 | Orthogonal to foliation within the Paleozoic basement | 8,4E-02 | 8,29E-14 | 43.09859375 N | 11.03340941 E |
| 16 | Orthogonal to foliation within the Paleozoic basement | 2,4E-03 | 2,37E-15 | 43.09859375 N | 11.03340941 E |
| 17 | Orthogonal to foliation within the Paleozoic basement | 4,9E-04 | 4,84E-16 | 43.09859375 N | 11.03340941 E |
|  |  | | | | |
| 1 | Parallel to foliation within the Paleozoic basement | 2,9E-03 | 2,86E-15 | 43.09867338 N | 11.0332757 E |
| 2 | Parallel to foliation within the Paleozoic basement | 1,2E-01 | 1,18E-13 | 43.09867338 N | 11.0332757 E |
| 3 | Parallel to foliation within the Paleozoic basement | 2,4E-02 | 2,37E-14 | 43.09867338 N | 11.0332757 E |
| 4 | Parallel to foliation within the Paleozoic basement | 2,4E-02 | 2,37E-14 | 43.09867338 N | 11.0332757 E |
| 5 | Parallel to foliation within the Paleozoic basement | 9,9E-02 | 9,77E-14 | 43.09867338 N | 11.0332757 E |
| 6 | Parallel to foliation within the Paleozoic basement | 3,7E-02 | 3,65E-14 | 43.09867338 N | 11.0332757 E |
| 7 | Parallel to foliation within the Paleozoic basement | 2,4E-01 | 2,37E-13 | 43.09867338 N | 11.0332757 E |
| 8 | Parallel to foliation within the Paleozoic basement | 1,4E-01 | 1,38E-13 | 43.09867338 N | 11.0332757 E |
| 9 | Parallel to foliation within the Paleozoic basement | 2,8E-01 | 2,76E-13 | 43.09867338 N | 11.0332757 E |
| 10 | Parallel to foliation within the Paleozoic basement | 2,1E-01 | 2,07E-13 | 43.09867338 N | 11.0332757 E |
| 11 | Parallel to foliation within the Paleozoic basement | 7,7E-01 | 7,60E-13 | 43.09867338 N | 11.0332757 E |
| 12 | Parallel to foliation within the Paleozoic basement | 9,2E-01 | 9,08E-13 | 43.09867338 N | 11.0332757 E |
| 13 | Parallel to foliation within the Paleozoic basement | 1,3E-01 | 1,28E-13 | 43.09867338 N | 11.0332757 E |
| 14 | Parallel to foliation within the Paleozoic basement | 3,0E-02 | 2,96E-14 | 43.09867338 N | 11.0332757 E |
